# −60 °C solution synthesis of atomically dispersed cobalt electrocatalyst with superior performance

**DOI:** 10.1038/s41467-019-08484-8

**Published:** 2019-02-05

**Authors:** Kai Huang, Le Zhang, Ting Xu, Hehe Wei, Ruoyu Zhang, Xiaoyuan Zhang, Binghui Ge, Ming Lei, Jing-Yuan Ma, Li-Min Liu, Hui Wu

**Affiliations:** 1 State Key Laboratory of New Ceramics and Fine Processing, School of Materials Science and Engineering, Tsinghua University, Beijing, 100084 China; 2 State Key Laboratory of Information Photonics and Optical Communications & School of Science, Beijing University of Posts and Telecommunications, Beijing, 100876 China; 3 Beijing Computational Science Research Center, Beijing, 100193 China; 4 State Key Joint Laboratory of Environment Simulation and Pollution Control, School of Environment, Tsinghua University, Beijing, 100084 China; 5 Institute of Physical Science and Information Technology, Anhui University, Hefei, China; 6 Beijing National Laboratory for Condensed Matter Physics, Institute of Physics, Chinese Academy of Science, Beijing, 100190 China; 7 Shanghai Synchrotron Radiation Facility, Shanghai Institute of Applied Physics, Chinese Academy of Science, Shanghai, 201204 China; 8 School of Physics, Beihang University, Beijing, 100191 China

## Abstract

Temperature can govern morphologies, structures and properties of products from synthesis in solution. A reaction in solution at low temperature may result in different materials than at higher temperature due to thermodynamics and kinetics of nuclei formation. Here, we report a low-temperature solution synthesis of atomically dispersed cobalt in a catalyst with superior performance. By using a water/alcohol mixed solvent with low freezing point, liquid-phase reduction of a cobalt precursor with hydrazine hydrate is realized at −60 °C. A higher energy barrier and a sluggish nucleation rate are achieved to suppress nuclei formation; thus atomically dispersed cobalt is successfully obtained in a catalyst for oxygen reduction with electrochemical performance superior to that of a Pt/C catalyst. Furthermore, the atomically dispersed cobalt catalyst is applied in a microbial fuel cell to obtain a high maximum power density (2550 ± 60 mW m^−2^) and no current drop upon operation for 820 h.

## Abstract (web summary)

Rapid nucleation and growth hinder the formation of atomically dispersed metals on solid supports in solution. Here the authors report a low-temperature solution-phase synthesis for isolated cobalt atoms on nitrogen-doped carbon, with electrocatalytic activity for oxygen reduction in a microbial fuel cell.

## Introduction

Solution-phase syntheses have been studied for hundreds of years for fabrication of solid-state materials, including metals and their compounds. An energy barrier is usually overcome for the reaction products to aggregate as precipitate, and the nuclei formation and growth represent the first stages undertaken by the product species^1–3^. The rapid nucleation and growth of solid-state reaction products hinder the formation of ultrafine nanocrystals or even atomically dispersed metals in solution. Therefore, effective suppression of nuclei formation in solution synthesis becomes extremely important, but remains a significant challenge. Tremendous research efforts have been devoted to control the structures of products in solution synthesis by adopting various approaches such as microfluidic engineering, surfactant-mediated approach, templated synthesis, and so on^4–7^. Increasing the energy barrier and reducing the kinetics for the nuclei formation in solution-phase reactions potentially provides an effective pathway to suppress the nanocrystal formation. The chemical reduction of metal cations (M^n+^) using reducing agents was considered as an example^8–10^, which can be represented as follows:

1 $$\mathrm{M}^{{n + }}{\mathrm{ + }}\mathrm{ne}^ - \to \mathrm{M}^0{.}$$

The products immediately aggregate to form metal nanoparticles in a typical solution-phase synthesis driven by high surface free energy of reduced metal atoms. Classically but instructively, the formation of final metal nanocrystals must flip over the energy gap for the nuclei formation (∆*G*_1_). The nuclei formation, which is driven by the thermodynamic free-energy difference between the two phases, requires the aggregation of atoms to overcome the nucleation barrier^9–11^, which is represented as follows:

2 $$\Delta {G}_1 = \frac{{{\mathrm{16\pi \gamma }}^3{\mathrm{\nu }}^2}}{{3{k}_{\mathrm{B}}^2{T}^2\left( {{\mathrm{ln}}\,{{S}}} \right)^2}}{,}$$

where *γ* is the increase in the free energy per unit surface area of the nucleus, *ν* is the molar volume of the nucleus, *k*_*B*_ is the Boltzmann constant, *T* is the temperature, and *S* is the supersaturation concentration of the solution. Obviously, temperature is a critical parameter to control ∆*G*_1_. In a certain solution reaction, with the maintained solution concentration (*C*), ∆*G*_1_ increases with the decrease in temperature. Notably, in principle, if we conduct the solution reaction at a relatively low temperature sufficient for the occurrence of the reaction (see Supplementary Note 1), but with the significantly increased nucleation barrier ∆*G*_1_ and critical nucleus size (Supplementary Fig. 1), the ability to regulate the nuclei formation should be attained which results in the formation of atomically dispersed products in solution. In addition, the suppressive nuclei formation also benefits from the decreased reduction rate and nucleation rate in an ultra-low temperature solution (also shown in Supplementary Note 1), providing opportunities for solution-phase synthesis at the very initial precipitation process.

Atomically dispersed catalysts have recently attracted tremendous attention due to their high performance for many reactions. Particularly, high-efficient, durable, and cost-effective catalysts for oxygen reduction reaction (ORR) play a key important role for widespread commercialization of fuel cells. Single-atom nonprecious metals have emerged as promising candidates for ORR; however, chemistry that enables rapid and facile synthesis of atomically dispersed catalysts with performance superior to expensive and scarce platinum (Pt)-based catalysts still faces major challenges. Therefore, we explore the low-temperature solution chemistry to synthesize high-performance (ORR) electrocatalysts composed with atomically dispersed nonprecious metals.

Herein, we report a low-temperature solution-phase synthesis approach to obtain isolated cobalt (Co) atoms on nitrogen (N)-doped mesoporous carbon (NMC) substrates as electrocatalysts for ORR. In contrast to the conventional solution syntheses under room temperature (RT) conditions, in this synthesis approach the temperature of chemical reduction of cobalt precursor in a water/alcohol mixed solvent system was considerably reduced down to −60 °C. Consequently, the regulated thermodynamics and kinetic changes ensured a completely suppressed nucleation of reduced Co atom species in solution and on NMC substrates during the synthesis. The experimental results demonstrated high ORR activity and long-term operational stability of the catalysts in both alkaline and neutral electrolyte solutions, with the maximum atom efficiency and most exposed active sites. Furthermore, we assembled a microbial fuel cell (MFC) using atomically dispersed Co in the cathode for ORR catalysis and achieved attractive cell performance for potential wastewater treatment and other waste-to-energy applications.

## Results

### Solution chemistry at −60 °C

A typical chemical reduction of Co^2+^ using hydrazine hydrate as a reducing agent is described as follows:

3 $$\begin{array}{l}{\mathrm{2CoCl}}_2{\mathrm{ + N}}_2{\mathrm{H}}_5{\mathrm{OH + 4KOH}} \to {\mathrm{2Co + 4KCl}}\\ {\mathrm{ + 5H}}_2{\mathrm{O + N}}_2\end{array}{.}$$

**Fig. 1:**
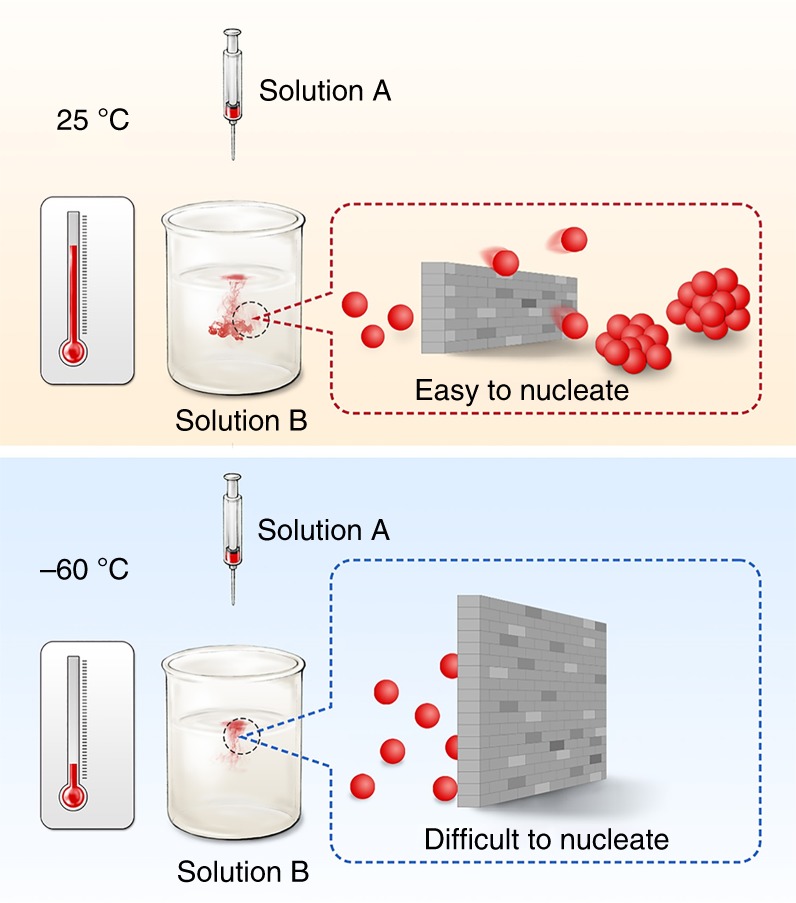
Schematic illustration of the ultra-low temperature solution reduction process. In our design, the nucleation of reduced metal atoms can be significantly suppressed mainly due to the sluggish thermodynamics and kinetic process at ultra-low temperature (bottom row). In contrast, the nuclei formation occurs easily at room temperature (upper row)

Eq. (3) represents the reduction reaction of Co^2+^ in alkaline solution under different temperatures. Noteworthy, the reaction is still thermodynamically favorable at −60 °C (as summarized in Supplementary Note 1 and Supplementary Table 1), indicating that the reduced Co can be obtained as product at −60 °C in principle. The temperature of the reaction system with liquid-phase environment was maintained at −60 °C using water/alcohol (1:9 in volume ratio) mixture as solvent system, with a freezing point of −78.5 °C. Figure 1 illustrates the formation of Co clusters or nanoparticles from the uncontrolled nucleation and growth at RT by conventional solution-phase reduction, and this result is in accordance with previous literature reports. However, solution synthesis at −60 °C leads to the formation of atomically dispersed Co-complex solution due to the occurrence of an extremely suppressive nucleation. It was considered that the increase in temperature of the solution to RT led to the disappearance of the high-energy gap and possible aggregation of the products to particles. Therefore, the reaction products were stabilized by mixing the solution with NMC without increasing the solution temperature back to RT, and the Co atomic species were absorbed on substrates at low temperatures by filter drying the solvents. After naturally drying at RT, fully anchored isolated Co atoms on NMC substrate (Co/NMC-LT, where LT denotes low temperature) were obtained compared to clustered Co species on NMC substrate (Co/NMC-RT) by solution synthesis at RT as identified by the aberration-corrected high-angle annular dark field-scanning transmission electron microscopy (HAADF-STEM) images (Supplementary Fig. 2). To further illuminate the significance of ultra-low temperature of −60 °C, sub-nano clusters and particles about tens of nanometer were also obtained at −30 and 60 °C (Supplementary Fig. 3). Moreover, we also achieved fully anchored isolated iron (Fe), silver (Ag), and platinum (Pt) atoms on NMC substrate by solution synthesis at −60 °C, as shown in Supplementary Fig. 4.

### Characterization of atomically dispersed cobalt atoms

**Fig. 2:**
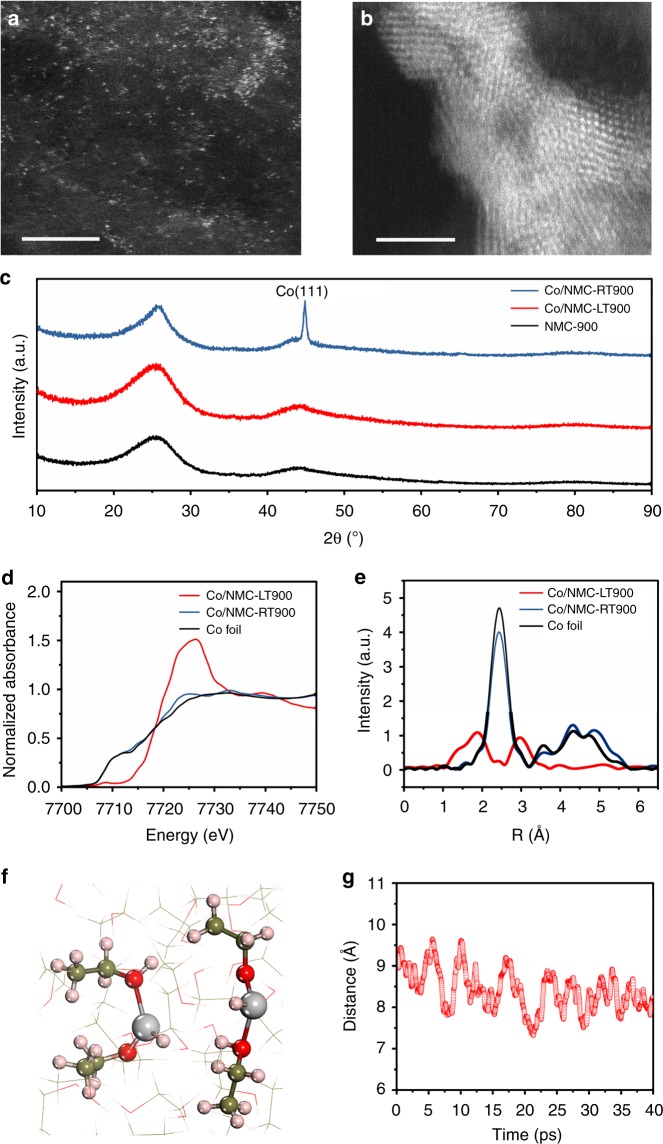
Characterization and simulation of different samples. High-angle annular dark field-scanning transmission electron microscopy (HAADF-STEM) images of **a** atomic Co catalyst (Co/NMC-LT900) and **b** Co clusters or nanoparticles catalyst (Co/NMC-RT900), scale bar: 2 nm. **c** X-ray diffraction (XRD) patterns of Co/NMC-LT900, Co/NMC-RT900, and annealed N-doped mesoporous carbon catalyst (NMC-900). **d** Normalized X-ray absorption near-edge structure (XANES) spectra at the Co K-edge and **e** Extended x-ray absorption fine structure (EXAFS) spectra for Co foil, Co/NMC-LT900, and Co/NMC-RT900. **f** Simulated atomic structures of Co atoms in solution, the ethanol molecules bound with Co atoms are highlighted by balls. The red, pink, olive, and silver balls or bonds represent O, H, C, and Co atoms, respectively. **g** The distance between two Co atoms as a function of time (40 ps) during the first-principle molecular dynamics (FPMD) calculation

The Co/NMC-LT sample was subsequently heated at 900 °C to activate the Co catalytic cites (designated as sample Co/NMC-LT900), which retained atomic dispersion of Co moieties (Fig. 2a and Supplementary Fig. 5a–d). However, clusters or nanoparticles phase of Co on Co/NMC obtained at RT (Co/NMC-RT900) can be observed at 900 °C due to the further crystallization of glomerate Co atoms (Fig. 2b and Supplementary Fig. 5e–h). This result was also confirmed by the X-ray diffraction analysis presented in Fig. 2c. No obvious characteristic peaks of Co can be observed for Co/NMC-LT900 samples. On the contrary, Co (111) peaks can be detected for the Co/NMC-RT900 sample. To further investigate the structure of Co species at the atomic level, X-ray absorption fine structure (XAFS) measurements were carried out. Figure 2d displays the Co K-edge X-ray absorption near-edge structure (XANES) spectra of Co foil, Co/NMC-LT900 and Co/NMC-RT900. The position of the white line peak for Co/NMC-LT900 is higher than Co foil which indicates the atomically dispersed Co atoms are positive charged. By contrast, the obtained XANES spectrum of Co/NMC-RT900 is very close to that of Co foil, revealling the dominance of metallic Co in Co/NMC-RT900. This observation agrees well with the XPS results in Supplementary Fig. 6, only Co-N_x_ moieties exist in Co/NMC-LT900 sample, however, one major components corresponding to metallic Co (778.5 eV) as well as oxidized Co species (780.2 eV) and Co–N_*x*_ moieties (781.6 eV) can be identified in Co/NMC-RT900^12,13^. According to the extended XAFS spectra in Fig. 2e and Supplementary Table 2, Co/NMC-LT900 exhibit a mean bond length about 1.98 Å which can be attributed to the Co–N/C first coordination shell, and no obvious Co–Co path (2.49 Å) like that of Co/NMC-RT900. This result furhter suggests that the Co atoms in Co/NMC-LT900 are atomically dispersed and coordinated with N/C structure on NMC substrate. Noteworthy, Co/NMC-LT900 and Co/NMC-RT900 exhibit a dominant pyridinic–N and graphitic–N components with no obvious difference in their relative amount, due to a total loss of pyrrolic–N occurred during high-temperature annealing (Supplementary Figs. 7, 8). Considering the different coordination conditions of Co species, chemically reduced Co atoms in solution at RT can go through a complex process of nucleation, agglomeration, crystallization, and coordination with N atoms for Co/NMC-RT900. Nonetheless, atomically dispersed Co obtained in solution phase at −60 °C is fully anchored and stabilized by Co–N_*x*_ coordinations.

### Electrocatalyst performance

**Fig. 3:**
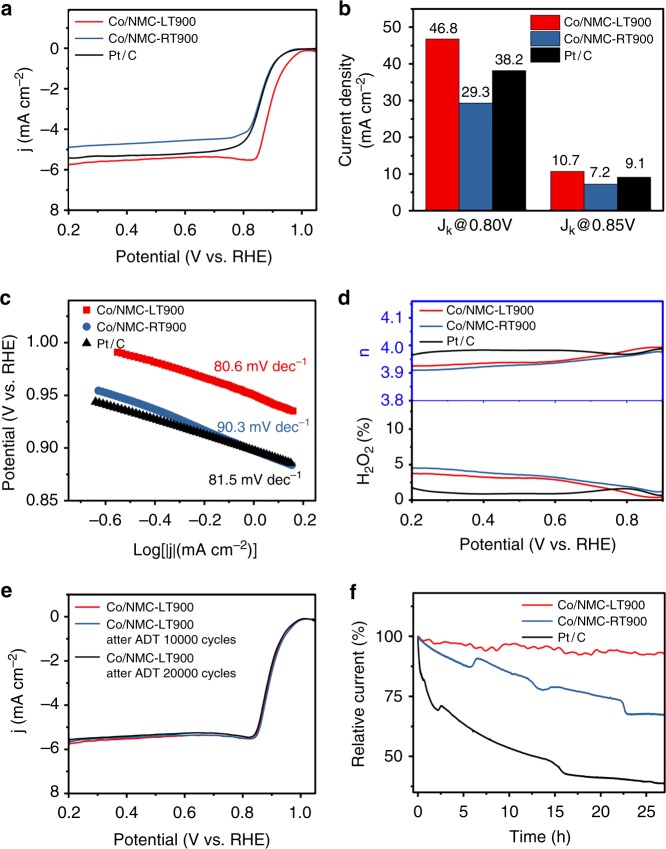
Electrocatalysis of oxygen reduction in O_2_-saturated alkaline electrolyte. **a** Rotating disk electrode (RDE) polarization curves of atomic Co catalyst (Co/NMC-LT900), Co clusters or nanoparticles catalyst (Co/NMC-RT900), and commercial platinum–carbon catalyst (Pt/C) in 0.1 M KOH solution at 1600 rpm calibrated with linear sweep voltammetry (LSV) data tested in N_2_. **b** Comparison of *J*_*k*_ values for different potential values. **c** Tafel slope values at low over-potential regions with sweep rate 10 mV s^−1^ and rotation rate 1600 rpm. **d** Electron transfer number *n* (top) and H_2_O_2_ yield (bottom) vs. potential. **e** RDE polarization curves of Co/NMC-LT900 before and after 10,000 and 20,000 potential cycles, calibrated with LSV data tested in N_2_. **f** Chronoamperometry (CA) curves of different catalysts at 0.85 V vs. RHE and rotation rate 1600 rpm

**Fig. 4:**
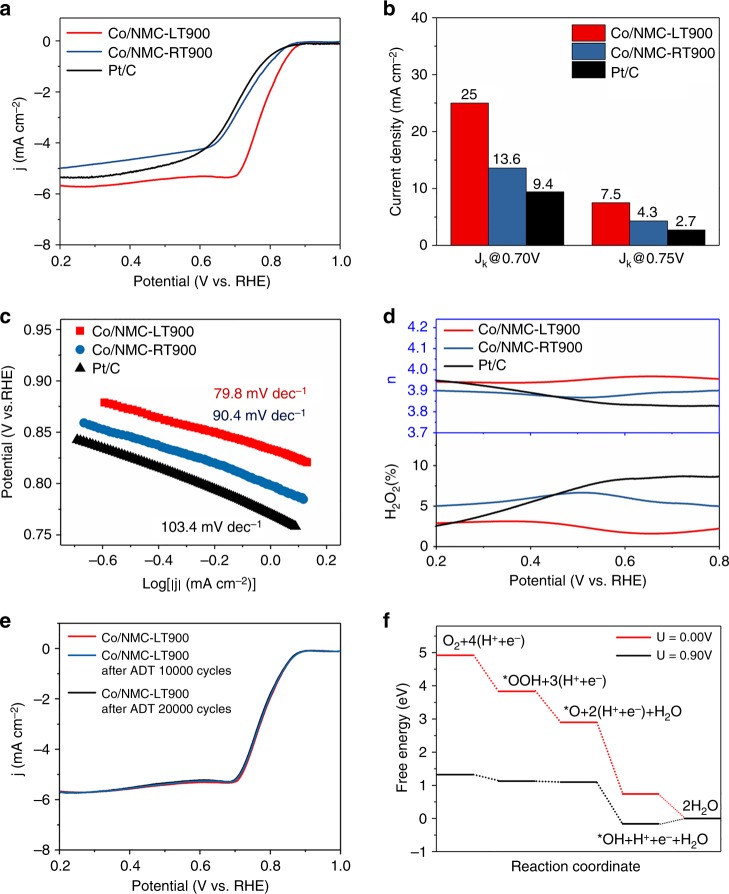
Electrocatalysis in O_2_-saturated near neutral electrolyte and free-energy calculation. **a** Rotating disk electrode (RDE) polarization curves of atomic Co catalyst (Co/NMC-LT900), Co clusters or nanoparticles catalyst (Co/NMC-RT900), and commercial platinum–carbon catalyst (Pt/C) in 0.05 M phosphate buffer solution (PBS) solution at 1600 rpm calibrated with linear sweep voltammetry (LSV) data tested in N_2_. **b** Comparison of *J*_*k*_ at different potential values. **b** Tafel slope values at low over-potential regions with sweep rate 10 mV s^−1^ and rotation rate 1600 rpm. **d** Electron transfer number *n* (top) and H_2_O_2_ yield (bottom) vs. potential. **e** RDE polarization curves of Co/NMC-LT900 before and after 10,000 and 20,000 potential cycles, calibrated with LSV data tested in N_2_. **f** Calculated free-energy profiles for oxygen reduction reaction (ORR) steps on Co/NMC-LT900, and *U* is the applied potential (vs. RHE) during the ORR process. Detailed experimental data are listed in Supplementary Tables 4 and 5

Development of high-efficient, stable, and durable nonprecious metals catalysts for ORR in fuel cells and metal–air batteries has been regarded as a critical issue in recent decades. We electrochemically characterized Co/NMC-LT900, Co/NMC-RT900, and Pt/C catalysts using a rotating disk electrode (RDE) and rotating ring-disk electrode (RRDE) in O_2_-saturated 0.1 M KOH solution. Co/NMC-RT900 exhibited an onset potential (*E*_*onset*_) of 0.968 V and a half-wave potential (*E*_1/2_) of 0.858 V, resembling with Pt/C catalyst. Comparative analyses indicated that the elevated *E*_*onset*_ and *E*_1/2_ values of Co/NMC-LT900 catalyst were 1.03 and 0.897 V, which were 62 and 39 mV more positive than that of Pt/C catalyst, respectively. As expected, the ORR current density of Co/NMC-LT900 also increased significantly (Fig. 3a). Higher kinetic current density (*J*_*k*_) at 0.85 V (46.8 mA cm^−2^) and 0.80 V (10.7 mA cm^−2^) and lower Tafel slope of 80.6 mV dec^−1^ further confirmed the prominent ORR activity of Co/NMC-LT900 (Fig. 3b, c). Supplementary Table 2 summarizes that Co/NMC-LT900 exhibits a surpassed ORR activity with respect to recently reported transition metals-based nanocatalysts and atomically dispersed nonprecious metals catalysts^14–18^, in terms of *E*_*onset*_, *E*_1/2_, *and J*_*k*_ (Supplementary Table 3). The high electron transfer rate and low-H_2_O_2_ yield (<5%) over the entire potential range derived from RRDE tests, indicated a direct four-electron ORR electrocatalytic pathway over Co/NMC-LT900 (Fig. 3d). The linear Koutecký–Levich (K–L) plots and near parallelism of the fitting lines for Co/NMC-LT900 obtained from RDE polarization tests by varying rotation rates, indicate first-order reaction kinetics toward the concentration of dissolved O_2_ and a potential-independent electron transfer rate (Supplementary Fig. 9a). The average electron transfer number (*n*) was calculated to be ~3.94 at 0.20–0.80 V from the slopes of K–L plots, which also indicates that Co/NMC-LT900 favors a four-electron oxygen reduction process. Figure 3e, f further display both typical accelerated durability tests (ADTs) and long-term chronoamperometry (CA) tests performed for the stability analysis. Compared to the degenerative ORR activity of Pt/C and Co/NMC-RT900 catalysts (Supplementary Fig. 9b–f), there was no obvious decay in *E*_1/2_ values and reduction current density for Co/NMC-LT900 catalysts after 20,000 continuous potential cycles and 27 h CA test operation at 0.85 V, indicating the extraordinary durability of Co/NMC-LT900 catalysts. Furthermore, Supplementary Fig. 10 exhibits a detailed comparison of ORR activity for Co/NMC-LT, Co/NMC-LT400, Co/NMC-LT800, pure NMC, and NMC-900 catalysts, which reveals that the high-electrocatalytic performance of Co/NMC-LT900 catalyst should be attributed to the formation of highly effective active sites by incorporating Co atoms with NMC at a suitable thermal activaton temperature of 900 °C as previously reported. Moreover, we obtained similar results in neutral O_2_-saturated phosphate buffer solution (PBS) solution (0.05 M) (Fig. 4 and Supplementary Fig. 11). The *E*_*onset*_ and *E*_1/2_ of Co/NMC-LT900 were, respectively, 20 and 85 mV more positive than that of Pt/C catalyst (0.885 V for *E*_*onset*_ and 0.694 V for *E*_1/2_). It also exhibited enhanced *J*_*k*_ values at 0.75 V (7.5 mA cm^−2^) and 0.70 V (25.0 mA cm^−2^), which were 2.78 and 2.66 times than that of Pt/C, respectively. A favorable kinetic process of Co/NMC-LT900 with respect to Pt/C in neutral solution was demonstrated (79.8 mV dec^−1^ vs. 103.4 mV dec^−1^). The high electron transfer rate, low-H_2_O_2_ yield (<2.5%), and average electron transfer number of 3.95 indicated the direct 4-electron oxygen reduction process of Co/NMC-LT900 in neutral electrolyte (Fig. 4d and Supplementary Fig. 11a). Moreover, Fig. 4e also demonstrates negligible decay in *E*_1/2_ and reduction current density for Co/NMC-LT900 after 20,000 continuous potential cycles during ADT, superior to Co/NMC-RT900 and Pt/C (Supplementary Fig. 11b,c). As shown in Supplementary Fig. 12 and Supplementary Table 4, the electrochemical impedance spectroscopy measurements under ORR reaction conditions in O_2_-saturated 0.1 M KOH solution and 0.05 M PBS solution. The Nyquist plots indicate that Co/NMC-LT900 exhibits the smallest charge–transfer resistance (*R*_ct1_) compared to those of Co/NMC-RT900and Pt/C, suggesting a fast charge–transfer rate of Co/NMC-LT900 electrocatalyst during the ORR.

Moreover, it is of significant importance to understand the high activity for M–N/C ORR catalysts for further rational and advanced design. Conventional high-temperature pyrolysis process has been well-developed for M–N/C catalysts or even isolated Fe or/and Co atoms on various N-doped carbon substrate catalysts^19–27^. However, various reactions (carbonization of nitrogenous carbon precursors and reactions of the metal moieties) always occur leading to the formation of N-doped carbon scaffolds with M–N_*x*_ sites, C–N_*x*_ sites, metallic M, and M_3_C nanoparticles, like the case of Co/NMC-RT900 in this study. By comparison, with a direct atomic-level synthesis in solution at −60 °C by suppressing nucleation, we demonstrated that chemically reduced Co species in solution coordinated with pyridinic–N on NMC substrates and then got converted into a more active Co–N_*x*_ sites embedded into the carbon skeleton by thermal activation^20,28,29^. To investigate the electrocatalytic activity of isolated Co atoms adsorbed on pyridinic–N, we further explored the ORR performance by computing the free-energy diagram of ORR. The stable adsorption structures of intermediate species (*OOH, *O, and *OH) on N-doped graphene are shown in Supplementary Fig. 13. Figure 4f demonstrates that the ORR process on Co adsorbed pyridinic–N is composed of four steps. With *U* = 0.90 V, all reaction steps are thermodynamically downhill except for the last uphill one (*OH desorption), which indicates that the first three ORR steps are exothermic and the final step involving *OH desorption is the rate-determining step. The adsorption energy of intermediate species and corresponding free-energy change (∆*G*) for each ORR steps are listed in Supplementary Table 5. It is obvious that the rate-determining step needs a very small energy of ~0.16 eV at *U* = 0.90 V, which is about 0.09 eV smaller than that of Pt/C (Supplementary Table 6)^30^. Consequently, the ORR electrocatalytic activity of Co/NMC-LT900 catalyst with atomically dispersed Co atoms adsorbed on pyridinic–N site can be superior to that of Pt/C catalyst.

### Full fuel cell performance

**Fig. 5:**
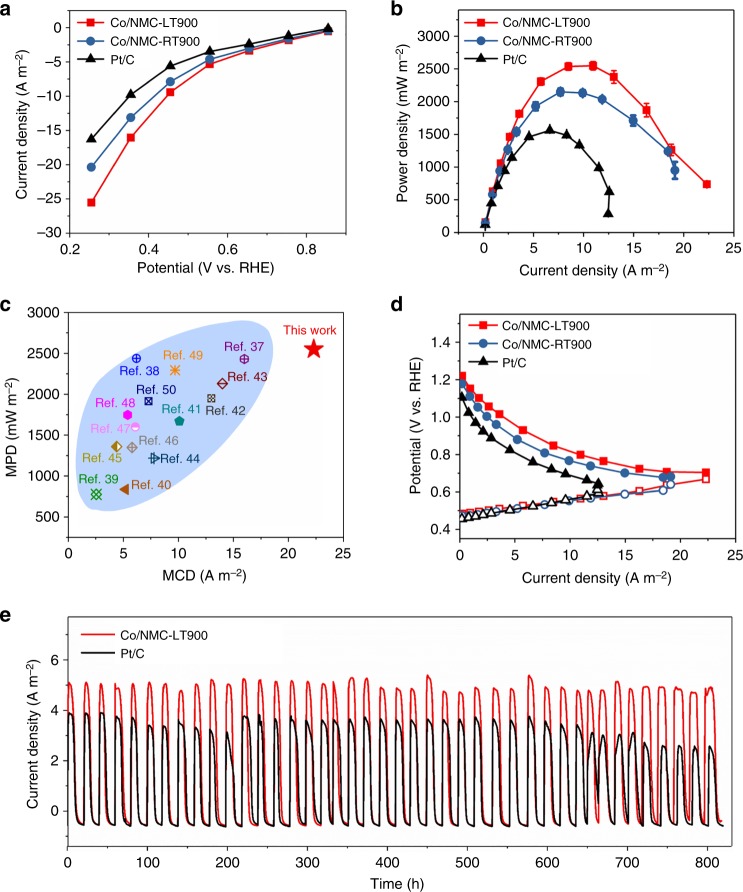
Microbial fuel cell performance. **a** Current–potential curves of different cathodes in abiotic electrochemical cells obtained by chronoamperometry (CA) tests. **b** Power densities as a function of current density. **c** Detailed comparison between of atomic Co catalyst (Co/NMC-LT900) and other state-of-the-art oxygen reduction reaction (ORR) catalysts for cathodes, in view of maximum power density (MPD) and maximum current density (MCD), including Fe–BP(N)^37^, Fe–N–C^38^, N–graphene^39^, CNT–texitile–Pt^40^, N–Fe/Fe_3_C@C^41^, Fe–Ricobendazole^42^, Fe–AAPyr-5^43^, 3D Fe–N–C^44^, Ag/FeS/PGC^45^, N-doped graphene^46^, N–CNT^47^, CNF_1000_^48^, Fe–N–C/AC^49^, and CoO_x_/CoP^50^. **d** Electrode potentials as a function of current density. **e** Current density generation trend recorded for a long-term cycling tests

To further validate the excellent ORR performance revealed by electrochemical analysis, MFCs with various air-cathodes were further assembled, which could convert chemical energy of mass-produced wastewater directly into indispensable electricity for potentially solving the energy and environmental problems simultaneously. Figure 5a shows the cathode polarization curves obtained by performing CA tests, illustrating that Co/NMC-LT900 and Co/NMC-RT900 display higher catalytic activity than Pt/C, despite identical loadings investigated and with the added benefit of a much lower cost. The Co/NMC-LT900 cathode showed the highest current density of 25.5 A m^−2^ at 0.255 V vs. RHE, which was 25% and 56% higher than that of Co/NMC-RT900 (20.4 A m^−2^) and Pt/C (16.3 A m^−2^), respectively. In general, various MFCs showed an open circuit voltage (OCV) in the following order: Co/NMC-LT900 (0.83 V) > Co/NMC-RT900 (0.80 V) > Pt/C (0.78 V) after operation without external resistance for 22 h (Supplementary Figs. 14 and 15). The highest OCV of Co/NMC-LT900-catalyzed MFCs also indicated the superior ORR activity, in accordance with the results from electrocatalytic tests. Moreover, power curves also confirmed the previously identified trend of the cathode polarization curves in clean conditions. The maximum power density (MPD) of MFCs with Co/NMC-LT900, Co/NMC-RT900, and Pt/C cathodes were 2550 ± 60, 2150 ± 57, and 1560 ± 15 mW m^−2^, respectively. Noteworthy, the MFC assembled with Co/NMC-LT900 cathode produced an excellent MPD, which was 119% and 163% of that from MFCs with Co/NMC-RT900 and Pt/C cathodes, respectively. Furthermore, the identical trend was also observed for the maximum current density (MCD), where Co/NMC-LT900 exhibited a remarkable value of 22.3 A m^−2^ (Fig. 5b). A detailed statistical analysis of MFC performance in terms of MPD and MCD was further conducted and the results are listed in Supplementary Table 7. The results confirmed that Co/NMC-LT900-based MFCs prevailed over the vast majority of other previously reported catalysts (Fig. 5c). Notably, the difference in power generation was only due to the performance of the cathode because the anodes performed nearly identically (Fig. 5d), thus verifying the superior ORR activity of the Co/NMC-LT900 cathode and the advantage of atomically distributed ORR active sites for MFC air-cathode catalysts in electricity production. Furthermore, the stability of various MFC systems was further evaluated in long-term operation (Fig. 5e). With a 50 Ω external resistance over 820 h, the current density of the MFC assembled with Co/NMC-LT900 cathode was well stable around 7.47 A m^−2^. Relatively, a distinct decrease from 5.98 to 4.98 A m^−2^ was found in the current density of the MFC assembled with Pt/C cathode due to the continuous attachment of active bacteria. Moreover, another obvious decrease from 5.80 to 4.24 A m^−2^ could still occur even when the biofilm was separated artificially to rebuild a relative higher current density at around 200 h. Therefore, the high stability of Co/NMC-LT900 can be attributed to the strong anti-contamination ability toward biofilms of atomically dispersed active sites. Notably, our results corroborated the prominent performance of Co/NMC-LT900 cathode ORR catalyst in practical MFC applications.

## Discussion

To investigate the stability of Co atoms both in solution and on NMC substrates, we first explored the atomic structure of atomically dispersed Co atoms in ethanol solvent by the first-principles molecular dynamics (FPMD) at −60 °C. As shown in Fig. 2f, each Co atom is surrounded with two ethanol molecules by forming bonds with oxygen atoms at a distance of ~2.1 Å, and one of the ethanol is dissociated by transferring the hydrogen of hydroxyl to the Co atom. During the 40 ps FPMD simulation (Fig. 2g), the two Co atoms are apart from each other with distances ranging from ~7.3 to ~9.6 Å, which means that the atomically dispersed Co atoms could be stable in solution. The possible nucleation process from atomically dispersed Co atoms to dimer was further examined by the metadynamics approach following the FPMD simulations^31,32^, where the final configuration of Co dimer is surrounded by four ethanol molecules and three of them are dissociated by transferring one hydrogen to Co atoms. The calculated free energy for the nucleation from two separated atomic Co to a dimer is about 0.24 eV as shown in Supplementary Fig. 16, although free-energy profile of the dimer state is not fully constructed in our metadynamic simulations. This result clearly suggests that the nuclei formation process can be effectively prevented at this ultra-low temperature, due to the contribution of ethanol molecules shell and further decreased kinetic process.

To further understand how the substrate affects the nucleation process, two further simulations were carried out by the first-principles calculations. First of all, we examined how the Co surrounded by the ethanol, as we predicted by the FPMD, interact with the N-doped graphene. Interestingly, the dissociated ethanol prefers to recover as molecular ethanol upon the Co surrounded by the ethanol absorbed on NMC substrate (Supplementary Fig. 17). Meanwhile this structure is about 0.30 eV more stable than the dissociated ethanol, such results clearly indicate that the substrate of N-doped graphene can greatly enhance the adsorption of Co, which can inhibit the nucleation in the ethanol as well. Then, we further examined whether the Co atoms have tendency to form dimer as exhibited in Supplementary Fig. 18. The corresponding results show that the dimer on the N-doped graphene is about 3.34 eV more unstable than the single Co on the N-doped graphene. Thus, the results further indicate that the substrate of N–graphene can help to anchor isolated Co atoms on graphene, while it prevents the nucleation on N–graphene. Moreover, the adsorption sites and energies of Co atoms on graphene with pyridinic–N and graphitic–N were further calculated. As shown in Supplementary Fig. 19, Co atom prefers to adsorb on carbon vacancy sites on the pyridinic–N, which leads to a small wrinkle of 0.62 Å. In case of graphitic–N, Co atom adsorbs on the hollow sites near the N atoms, and the surface remains flat. Moreover, the adsorption energies of Co atoms on pyridinic–N and graphitic–N were about −6.52 and −2.19 eV, respectively, revealing that Co atoms adsorbed on pyridinic–N was more thermodynamically stable. This result is consistent with previous reports that pyridinic–N could help to anchor transition metal atoms^33,34^.

In summary, we demonstrated that atomically dispersed cobalt (Co) with a total concentration of 2 mM could be obtained and stabilized in solution synthesis at −60 °C. By decreasing the reaction temperature, the energy barrier and rate of reduced metal atoms to form nuclei can be effectively regulated. High-loading Co atoms (4.66%) anchored on N-doped mesoporous carbon (NMC) substrates by N-coordinations exhibited excellent thermal stability at 900 °C, forming superior non-noble metal catalysts (Co/NMC-LT900) with atomically dispersed CoN_*x*_/C active sites for ORR. Moreover, we obtained better microbial fuel cell performance with Co/NMC-LT900 air-cathodes relative to the most recently reported ORR catalysts. We believe that this novel ultra-low temperature solution synthesis strategy will not only deepen the understanding of solid nucleation and growth of wet-chemistry reactions, but also pave the way for large-scale preparation of atomic and sub-nanometer materials with diverse potential applications.

## Methods

### General information

Except otherwise noted, all chemicals were purchased and used without purification. Additional details of materials and diversity of characterization techniques used are presented in the Supplementary Note 2.

### Synthesis of atomic and clusters or nanoparticles of cobalt samples

To maintain solution environment under ultra-low temperature conditions at −60 °C, a mixed solvent system consisting of ultrapure water and absolute ethanol was selected for any dissolution or dispersion process with a volumetric proportion of 1:9, respectively. For Co/NMC-LT900, 5 mL of CoCl_2_ solution A (0.01 M) was added dropwise into 20 mL of N_2_H_5_OH alkaline solution B (5.0 M) with the addition of KOH (0.05 M). The injection rate was controlled to be 0.25 mL min^−1^ by a syringe pump system. The mixed solution was then further allowed to react for another 2 h to yield atomically dispersed Co-based solution with a final metal species concentration of about 2 mM. Fully anchored atomic Co on NMC (Co/NMC-LT) was achieved by mixing atomically dispersed Co solution with NMC dispersion C under continuous stirring at −60 °C for another 5 h. This was followed by rinsing and collecting by vacuum filtration at −60 °C and naturally drying at RT. A general annealing process was adopted to obtain the thermally activated atomically dispersed Co on NMC catalyst (Co/NMC-LT900). Co/NMC-LT powder was placed in a tube furnace, and then heated to 900 °C for 1 h at a heating rate of 10 °C min^−1^ under flowing Ar gas (400 mL min^−1^) before allowing it to naturally cool down to RT. Thermally activated Co clusters or nanoparticles on NMC catalyst (Co/NMC-RT900) were also prepared by following the procedures similar to those for preparing Co/NMC-LT900, except that the solution temperature of liquid reduction reaction was kept at RT, i.e., 25 °C.

### Electrochemical and microbial fuel cell measurements

Details for ORR electrocatalytic analysis of various catalysts in this manuscript as well as the fabrication and electrochemical tests of air-cathodes are presented in Supplementary Information. Different air-cathodes were assembled in MFC reactors to evaluate the performance of ORR, power generation, and stability, with the diffusion layers facing the air. The anode was a graphite fiber brush (2.5 cm in both diameter and length) with a core of two twisted titanium wires that functioned as a current collector. All MFCs were inoculated with the effluent of well-developed MFCs for over 1 year to inoculate the anaerobic electrochemically active bacteria onto the anode. The synthetic wastewater medium consisted of sodium acetate (1 g L^−1^) in PBS (50 mM) mixed with minerals (12.5 mL L^−1^) and vitamins (5 mL L^−1^)^35^. All the MFCs were operated in fed-batch mode and in duplicate with a 50 Ω external resistance at a temperature of 30 ± 1 °C. To evaluate the durability performance, all MFCs were operated for 200 h, recording the current density variation with time.

Voltage (*U*) was recorded across an external resistance (*R*) every 20 min using a multimeter with a computerized data acquisition system (model 2700, Keithley Instruments, USA). Polarization curves were collected by a multicycle method by varying the external resistance from 5000 to 2 Ω, with each resistance being used for 20 min^36^. Furthermore, the OCV was measured without external resistance for 22 h. Current densities (*J*) and power densities (*P*) were normalized by the air-cathode projected area (*A* = 7 cm^2^), using *J* *=* *U/RA* and *P* *=* *JU*.

## Supplementary information

Supplementary Information

## Data Availability

All data are available from the authors upon reasonable request.
