# Supplementary material for: −60 °C solution synthesis of atomically dispersed cobalt electrocatalyst with superior performance
Source: Nat Commun. 2019 Feb 5;10:606. doi: 10.1038/s41467-019-08484-8 (PMC6363747; doi:10.1038/s41467-019-08484-8)
Supplement: Supplementary file 1 — Supplementary Information [file 41467_2019_8484_MOESM1_ESM.pdf]

Supplementary Materials for

-60 °C Solution Synthesis of Atomically Dispersed Cobalt Electrocatalyst with Superior Performance

Kai Huang[1,2†], Le Zhang[3†], Ting Xu[4†], Hehe Wei[1], Ruoyu Zhang[1], Xiaoyuan Zhang[4*], Binghui Ge[5,8*], Ming Lei[2], Jing-Yuan Ma[6], Li-Min Liu[7,3*], Hui Wu[1*]

[†]These authors contributed to this work equally.

[*]Correspondence to: huiwu@tsinghua.edu.cn (H.W.), liminliu@buaa.edu.cn (L.L.), zhangxiaoyuan@tsinghua.edu.cn (X.Z.) and bhge@iphy.ac.cn (B.G.).

Contents:

Supplementary Notes 1 to 2

Supplementary Figures 1 to 19

Supplementary Tables 1 to 7

Supplementary References 1 to 82

Supplementary Note 1

1. Thermodynamic calculation of solution reduction reaction

The fundamental criterion of reaction spontaneity (thermodynamic feasibility) ΔG can be described as follows:

$$\Delta G = \Delta H - T\Delta S \quad (1)$$

where S is the entropy, H is the enthalpy, and T is the solution temperature. Solution reactions occur spontaneously if ΔG is still negative at a relatively low temperature.

$$2CoCl_2 + N_2H_5OH + 4KOH \rightarrow 2Co + 4KCl + 5H_2O + N_2 \quad (2)$$

According to parameters listed in Supplementary Table 1, the Gibbs free energy changes in solution reduction reaction (2) can be calculated as follows:

$\Delta G_{-60\ ^oC}$ = -157.56 (kJ mol^{-1}) - (-60+273) × 618.61 (J mol^{-1}) = -289.3 kJ mol^{-1} < 0 and $\Delta G_{25\ ^oC}$ = -157.56 (kJ mol^{-1}) - (25+273) × 618.61 (J mol^{-1}) = -341.9 kJ mol^{-1} < 0, which indicates that the solution reduction reaction in equation (2) is still thermodynamically favorable at -60 ^{o}C. We also confirmed that the solution reduction reactions shown in the following equations (3-5) are still thermodynamically favorable at -60 ^{o}C.

$$2FeCl_2 + N_2H_5OH + 4KOH \rightarrow 2Fe + 4KCl + 5H_2O + N_2 \quad (3)$$

$\Delta G_{-60\ ^oC}$ = -381.59 (kJ mol^{-1}) - (-60+273) × 793.80 (J mol^{-1}) = -550.7 kJ mol^{-1} < 0, and $\Delta G_{25\ ^oC}$ = -381.59 (kJ mol^{-1}) - (25+273) × 793.80 (J mol^{-1}) = -618.1 kJ mol^{-1} < 0

$$2AgNO_3 + 2N_2H_5OH \rightarrow 2Ag + 2NH_4NO_3 + 2H_2O + N_2 \quad (4)$$

$\Delta G_{-60\ ^oC}$ = -1149.0 (kJ mol^{-1}) - (-60+273) × 256.12 (J mol^{-1}) = -1202.5 kJ mol^{-1} < 0, and $\Delta G_{25\ ^oC}$ = -1149.0 (kJ mol^{-1}) - (25+273) × 256.12 (J mol^{-1}) = -1225.3 kJ mol^{-1} < 0

$$H_2PtCl_6 + N_2H_5OH \rightarrow Pt + 6HCl + H_2O + N_2 \quad (5)$$

$\Delta G_{-60\ ^{\circ}C}$ = -671.13(kJ mol^{-1})-(-60+273)×285.13(J mol^{-1}) = -731.9 kJ mol^{-1} < 0, and $\Delta G_{25\ ^{\circ}C}$ = -671.13(kJ mol^{-1})-(25+273)×285.13(J mol^{-1}) = -756.1 kJ mol^{-1} < 0.

2. Temperature-dependent reduction rate

Recently, many groups have devoted extensive research efforts to search for a quantitative knob that can be adjusted to precisely manipulate the nucleation and growth of nanocrystals in a predictable manner. Some results have clearly demonstrated that the reduction rate of salt precursor not only plays an essential role in determining the outcome of a synthesis, but also serves as a quantitative knob for controlling the products of synthesis. In most concerned cases by supplying the reducing agent in great excess relative to the salt precursor, the reduction rate can be simplified to that of a pseudo-first-order reaction as follows:

$$\text{Rate}=k\left[M^{n+}\right]=Ae^{-E_a/RT}\left[M^{n+}\right] \tag{6}$$

where *k* is the rate constant, $\left[M^{n+}\right]$ corresponds to the concentrations of the salt precursor, *A* is the frequency factor, *R* is the universal gas constant, and *T* is the reaction temperature [1–5.] Obviously, the reduction rate decreases dramatically with the decrease in the reaction temperature, provided all other conditions remain unchanged. This indicates that the reaction temperature can be regarded as a key parameter to regulate and control the solution reduction process of metal precursors.

3. Temperature-dependent nucleation rate

A rate of nucleation of N particles during time *t* can be described by using an Arrhenius type equation (7) as follows:

$$\frac{dN}{dt}=A\exp\left(-\frac{16\pi\gamma^3v^2}{3k_B^3T^3(\ln S)^2}\right) \tag{7}$$

where A is a pre-exponential factor, γ is the increase in the free energy per unit surface area of the nucleus, v is the molar volume of the nucleus, k_B is the Boltzmann constant, *T* is the temperature, *S* is the supersaturation concentration of the solution. Temperature as one of key experimental

parameters can cause a dramatic decrease in the nucleation rate from RT to -60 ^{o}C, as reported by Kwon and Hyeon [6].

Supplementary Note 2

1. Materials

Cobalt(II) chloride hexahydrate ($CoCl_2$ $6H_2O$, 99.998% Co metal basis, Alfa Aesar), hydrazine hydrate (N_2H_5OH, 98%, Alfa Aesar), potassium hydroxide (KOH, 85%, Sinopharm Chemical Reagent), absolute ethanol (C_2H_5OH, 99.8%, Aladdin), NMC powders (8.0 at.% Nitrogen, XFNANO), carbon black powder (Vulcan XC-72, Cabot corporation, USA), polytetrafluoroethylene (PTFE, 60 wt.%, DuPont), and stainless steel mesh (type 304, McMasterCarr, USA) were used as received without any further purification.

2. Preparation of ultralow-temperature solution or dispersion

To maintain solution environment under ultralow-temperature conditions at -60 ^{o}C, a mixed solvent system consisting of ultrapure water and absolute ethanol was selected for any dissolution or dispersion process with a volumetric proportion of 1:9, respectively. For instance, Co-based metal precursor solution A (0.01 M) was obtained by dissolving $CoCl_2$ $6H_2O$ powders; diluted N_2H_5OH alkaline solution B (5.0 M) was prepared with the addition of KOH powder (0.05 M); and NMC dispersion C (2.5 mg mL^{-1}) was also formed by ultrasonic and stirring treatment.

3. Preparation of atomically dispersed cobalt solution

Atomically dispersed Co solution was prepared by an ultralow-temperature liquid reduction reaction process at -60 ^{o}C. Solution A (5 mL), solution B (20 mL), and dispersion C (20 mL) was first cooled down to -60 ^{o}C under stirring with an average freezing rate of -2 ^{o}C min^{-1} and then held for another 30 min. To trigger chemical reduction reaction, solution A was added dropwise into solution B with an injection rate of 0.25 mL min^{-1} controlled by a syringe pump system. The

mixed solution was then further allowed to react for another 2 h to yield atomically dispersed Co-based solution with a final metal species concentration of about 2 mM.

4. Preparation of atomically dispersed cobalt on nitrogen-doped mesoporous carbon catalyst (Co/NMC-LT900)

Fully anchored atomic Co on NMC (Co/NMC-LT) was achieved by mixing atomically dispersed Co solution with NMC dispersion C under continuous stirring at -60 ^{o}C for another 5 h. This was followed by rinsing and collecting by vacuum filtration at -60 ^{o}C and naturally drying at room temperature. A general annealing process was adopted to obtain the thermally activated atomically dispersed Co on NMC catalyst (Co/NMC-LT900). Co/NMC-LT powder was placed in a tube furnace, and then heated to 900 ^{o}C for 1 h at a heating rate of 10 ^{o}C min^{-1} under flowing Ar gas (400 mL min^{-1}) before allowing it to naturally cool down to room temperature.

5. Preparation of cobalt clusters or nanoparticles on nitrogen-doped mesoporous carbon catalyst (Co/NMC-RT900)

Thermally activated Co clusters or nanoparticles on NMC catalyst (Co/NMC-RT900) were also prepared by following the procedures similar to those for preparing Co/NMC-LT900, except that the solution temperature of liquid reduction reaction was kept at room temperature, i.e., 25 ^{o}C.

6. Preparation of thermally activated NMC catalyst (NMC-900)

For comparison, pure NMC was also annealed at 900 ^{o}C for 1 h under flowing Ar gas to form NMC-900 as a metal-free catalyst.

7. X-ray diffraction characterizations

Powder XRD patterns were acquired at room temperature using an X-ray diffractometer (D/max 2500V) at an operating voltage and current of about 40 kV and 150 mA, respectively. A typical 2θ scan range of 10-90° and a scanning speed of 8° min^{-1} were employed.

8. High-angle annular dark field-scanning transmission electron microscopy characterizations

Aberration-corrected HAADF-STEM images were acquired using a JEM-ARM200F transmission electron microscope operated at 200 kV.

9. X-ray photoelectron spectroscopy characterizations

XPS measurements were obtained using an X-ray photoelectron spectrometer (Escalab 250Xi) equipped with an Al Kα radiation source (1487.6 eV) and hemispherical analyzer with pass energy of 30.0 eV and an energy step size of 0.05 eV. The binding energy of the C 1s peak at 284.8 eV was considered as an internal reference. Spectral deconvolution was performed by Shirley background subtraction by using a Voigt function convoluting the Gaussian and Lorentzian functions.

10. Inductively coupled plasma-mass spectrometry characterizations

Inductively coupled plasma-mass spectrometry (ICP-MS, ELAN DRC-e) measurements were obtained to determine the final Co loading contents on NMC. The ICP-MS results confirmed that the Co metal loading of Co/NMC-LT900 and Co/NMC-RT900 were 4.66 and 4.72%, respectively.

11. XAFS experiments and data processing

XAFS measurements at the Co K-edge (7709 eV) in both transmission (for Co foil) and fluorescence (for samples) mode were performed at the BL14W1 in Shanghai Synchrotron Radiation Facility (SSRF) [7]. The electron beam energy was 3.5 GeV and the stored current was 260 mA (top-up). A 38-pole wiggler with the maximum magnetic field of 1.2 T inserted in the straight section of the storage ring was used. XAFS data were collected using a fixed-exit double-crystal Si(111) monochromator. A Lytle detector was used to collect the fluorescence signal, and the energy was calibrated using Co foil. The photon flux at the sample position was 2.1×10^{12} photons per second.

The raw data analysis was performed using IFEFFIT software package according to the standard data analysis procedures [8]. The spectra were calibrated, averaged, pre-edge background subtracted, and post-edge normalized using Athena program in IFEFFIT software package. The Fourier transformation of the k^3-weighted EXAFS oscillations, $k^3\chi(k)$, from k space to R space was performed over a range of 3.0-11.5 $Å^{-1}$ (3.0-14.2 for Co foil) to obtain a radial distribution function. And data fitting was done by Artemis program in IFEFFIT.

12. Air-cathode material and its fabrication

Air cathodes contain three layers including a catalyst layer, a current collector, and a diffusion layer. Carbon black powder, PTFE dispersion, and ethanol were mixed to obtain a diffusion layer onto a stainless steel mesh (60 × 60) by a press process according to previous study [9]. For Co/NMC-LT900, Co/NMC-RT900, and NMC-900, 60 mg samples were used as catalysts for air cathodes (11 cm^2 total area and 7 cm^2 projected area), leading to a final catalyst loading of 5.45 mg cm^{-2}. The catalyst layer was fabricated by coating the mixture of catalyst (60 mg), deionized water (388 μL), and PTFE dispersion (70 μL) onto the other side of stainless steel mesh in diffusion layer. Then another stainless steel mesh (60 × 60) facing the catalyst layer was pressed together at 10 MPa for 10 min and dried at 80 ^oC to assemble the final cathodes prior to use. Pt-based air-cathode was prepared by following the same procedure using 10% Pt on Vulcan XC-72 powder as a benchmark.

13. Electrochemical measurements

The electrochemical performance of air-cathodes was evaluated using a potentiostat (PGSTAT 128N, Metrohm Autolab, Netherlands) in a cube-shaped abiotic electrochemical reactor constituted by an anode cylindrical chamber (4 cm in length), and a cathode cylindrical chamber (2 cm in length) bolted together with an anion exchange membrane (AEM; AMI-7001, Membrane

International Inc., USA) in the middle. The inner diameter of cylindrical chamber was 3 cm, leading to a 28 mL total volume and 14 mL total volume for a two-electrode chamber. A high purity Pt mesh (99.99%, 1 cm^2) was placed in the anode chamber as the counter electrode, the cathode was placed on one side of the other chamber facing the air as a working electrode, and a saturated calomel reference electrode (SCE, +0.242 V vs. standard hydrogen electrode) was placed close to the working electrode, air-cathodes. Phosphate buffer solution (50 mM, PBS) was introduced as the electrolyte, which contained $NaH_2PO_4 \cdot H_2O$ (2.45 g L^{-1}), Na_2HPO_4 (4.57 g L^{-1}), NH_4Cl (0.31 g L^{-1}), and KCl (0.13 g L^{-1}). CA tests were performed by setting a potential in a stepwise manner after the reactor operated in open circuit condition for 3 h. Each potential (0.2, 0.1, 0, -0.1, -0.2, -0.3, and -0.4 V vs. SCE) was applied for 30 min. All potentials applied in this study were calibrated to the reversible hydrogen electrode (RHE) using the following equation: $E_{RHE} = E_{SCE} + 0.242 + 0.059 \times \text{pH}$.

All classical electrocatalytic tests were conducted in a conventional three-electrode electrochemical system containing 0.1 M KOH or 0.05 M PBS solution at room temperature using an Autolab PGSTAT-204 potentiostat equipped with the Nova 1.11 software. A rotating-disk glassy-carbon (area 0.196 cm^2) electrode coated with the catalyst ink served as the working electrode, an Ag/AgCl (3 M KCl, +0.214 V vs. standard hydrogen electrode) and a Pt mesh electrode were used as a reference and a counter electrode, respectively. Working electrode was prepared by the following procedure: catalyst (4 mg) was dispersed in a mixture of isopropyl alcohol (500 μL), water (460 μL), and Nafion solution (40 μL, 5%) for 20 min to form homogeneous catalyst inks. Then a certain volume of the catalyst ink was pipetted onto the GC surface with the nonprecious catalyst loading of 0.408 mg cm^{-2} and the loading of Pt/C (20%) was 0.102 mg cm^{-2}. After cyclic voltammetry (CV) activation for 30 cycles with a scan rate of 50 mV

s^{-1} in N_2-saturated electrolytes, RDE tests were performed in O_2-saturated solution at different rotation rates from 3600 to 900 rpm with a sweep rate of 10 mV s^{-1} at room temperature. The ADTs of the nonprecious catalysts and Pt/C were performed at RT by applying potential cycling in the O_2-saturated electrolyte at a sweep rate of 100 mV s^{-1} for 10,000 cycles and 20,000 cycles. It's important to note that we have also conducted the RDE test in N2 to subtract the corresponding capacitance current density for better comparison, as shown in Supplementary Figure 19 and 20. The potential range was 0.6-1.0 V vs. RHE in 0.1 M KOH and 0.5-0.9 V vs. RHE in 0.05 M PBS solution. Additional CA test was carried out at a constant voltage of 0.85 V vs. RHE in 0.1 M O_2-saturated KOH for 27 h. The electron transfer number (n) and kinetic current density (J_k) were calculated by using the K-L equation as follows:

$$\frac{1}{J}=\frac{1}{J_K}+\frac{1}{J_L}=\frac{1}{J_K}+\frac{1}{0.2nFCD^{2/3}v^{-1/6}\omega^{1/2}} \quad (8)$$

where J is the measured current density, J_K and J_L are the kinetic and limiting current densities, respectively, ω is the rotation speed (rpm) of the disk, n is the electron transfer number, F is the Faraday constant (96485 C mol^{-1}), C is the bulk concentration of O_2 (1.2×10^{-6} mol cm^{-3}), D is the diffusion coefficient of O_2 (1.9×10^{-5} cm^2 s^{-1}), and v is the kinematic viscosity of the electrolyte (0.01 cm^2 s^{-1}). RRDE measurements (glassy-carbon disk area of 0.2475 cm^2) were also performed using a CHI760E electrochemical workstation with a fixed ring electrode potential of 1.25 V vs. RHE, to monitor the H_2O_2 yield (H_2O_2%) and the electron transfer number (n) by using the following equations:

$$H_2O_2(\%)=200\times\frac{\frac{I_R}{N}}{\frac{I_R}{N}+I_D} \quad (9)$$

$$n=4\times\frac{I_D}{\frac{I_R}{N}+I_D} \quad (10)$$

where I_D is the disk current, I_R is the ring current, and $N = 0.4$ is the ring collection efficiency. All potentials applied herein were calibrated to the RHE using the following equation: $E_{RHE} = E_{Ag/AgCl} + 0.214 + 0.059 \times pH$.

14. Microbial fuel cell experiments

Different air-cathodes were assembled in MFC reactors to evaluate the performance of ORR, power generation, and stability, with the diffusion layers facing the air. The anode was a graphite fiber brush (2.5 cm in both diameter and length) with a core of two twisted titanium wires that functioned as a current collector. All MFCs were inoculated with the effluent of well-developed MFCs for over one year to inoculate the anaerobic electrochemically active bacteria onto the anode. The synthetic wastewater medium consisted of sodium acetate (1 g L^{-1}) in PBS (50 mM) mixed with minerals (12.5 mL L^{-1}) and vitamins (5 mL L^{-1}) [10]. All the MFCs were operated in fed-batch mode and in duplicate with a 50 Ω external resistance at a temperature of 30 ± 1 ^{o}C. To evaluate the durability performance, all MFCs were operated for 200 h, recording the current density variation with time.

Voltage (U) was recorded across an external resistance (R) every 20 min using a multimeter with a computerized data acquisition system (model 2700, Keithley Instruments, USA). Polarization curves were collected by a multicycle method by varying the external resistance from 5000 to 2 Ω, with each resistance being used for 20 min [11]. Furthermore, the OCV was measured without external resistance for 22 h. Current densities (J) and power densities (P) were normalized by the air cathode projected area ($A = 7$ cm^2), using $J = U/ RA$ and $P = JU$.

15. Computational Method

Calculations were performed by using the density functional theory (DFT) with Vienna ab initio package (VASP) [12,13]. The general gradient approximation of Perdew-Burke-Ernzerhof (GGA-

PBE) functional was used to describe the exchange-correlation interactions between electrons [14,15]. The energy cutoff was set at 500 eV for plane wave functions. In this study, the vacuum layers were set at 12 Å, and the van der Waals (vdW) interaction was calculated by the DFT-D3 method. The reciprocal space was sampled using a 2 × 2 × 1 point grid by using Monkhorst-Pack K-points scheme [16,17]. The structures were relaxed until the residual force on each atom was less than 0.01 eV $Å^{-1}$. In the calculations, a 10 × 10 × 1 graphene supercell was used. The pyridinic-N and graphitic-N are two typical NMC in the experiment; therefore, they were mainly considered in this study.

The adsorption energy (E_a) was calculated by using the following equation:

$$E_a = E_{substrate+adsorbate} - E_{substrate} - E_{adsorbate} \quad (11)$$

where $E_{substrate+adsorbate}$ is the total energy of adsorbate on substrate, $E_{substrate}$ is the total energy of substrate, and $E_{adsorbate}$ is the energy of the adsorbate. As for the Co adsorption on graphene, the total energy of single Co atom was used for the adsorbate during calculating the adsorption energy. Based on this definition, the smaller adsorption energy indicates the more stable adsorption on substrates. The ORR in alkaline medium is a complete $4e^-$ process, where the changes of free energy for each step were calculated to reflect the ORR activity. For every one electron transfer step, the free energy change (ΔG) can be expressed as follows:

$$\Delta G = \Delta E + \Delta ZPE - T\Delta S - eU \quad (12)$$

The ΔE, ΔZPE, TΔS, e, and U represent the energy changes, zero-point energy correction, entropic energy, the elementary charge, and the potential used during the ORR, respectively. The ΔE and ΔZPE values were obtained from DFT calculations and the TΔS was obtained from the standard thermodynamic data. The ORR process in this study includes the following four steps: $* + O_2 + H^+ + e^- \rightarrow *OOH$, $*OOH + H^+ + e^- \rightarrow *O + H_2O$, $*O + H^+ + e^- \rightarrow *OH$, and $*OH + H^+ + e^- \rightarrow$

H_2O. Here, the * and *OOH (*O and *OH) represent an adsorbed site of substrate and adsorbed OOH (O and OH), respectively. As shown in the main context, the OH desorption is the key step. In our calculations, we considered the ORR processes with one and two OH adsorption on the Co. The results show that the two OH adsorption on the Co exhibits the smaller energy barrier for ORR, as reported in the main context.

In order to investigate the stability of atomically dispersed Co in solution, the first-principle molecular dynamics (FPMD) simulation was carried with NVT ensemble along with a Nose-Hoover thermostat using CP2K/QUICKSTEP package [17], at the target temperatures of -60 ^{o}C. The FPMD simulations was carried out 40 ps. To reduce the basis set superposition error, the Gaussian functions with molecularly optimized double-ζ polarized basis sets (m-DZVP) were used [18]. Moreover, core electrons were described with norm-conserving Goedecker, Teter, and Hutter (GTH) pseudopotentials to expand wave functions of valence electrons [19]. The metadynamics simulations was first carried with FPMD with NVT for 60 ps and then with the Always Stable Predictor Corrector (ASPC) method for 73 ps [20]. The free energy profile was constructed after the metadynamics simulations. Here the distance between Co and Co atoms was used for the collective variables. During the simulations, each 100 steps, one new hill was added. The Gaussian height and width are 0.0054 eV and 0.20 eV, respectively.

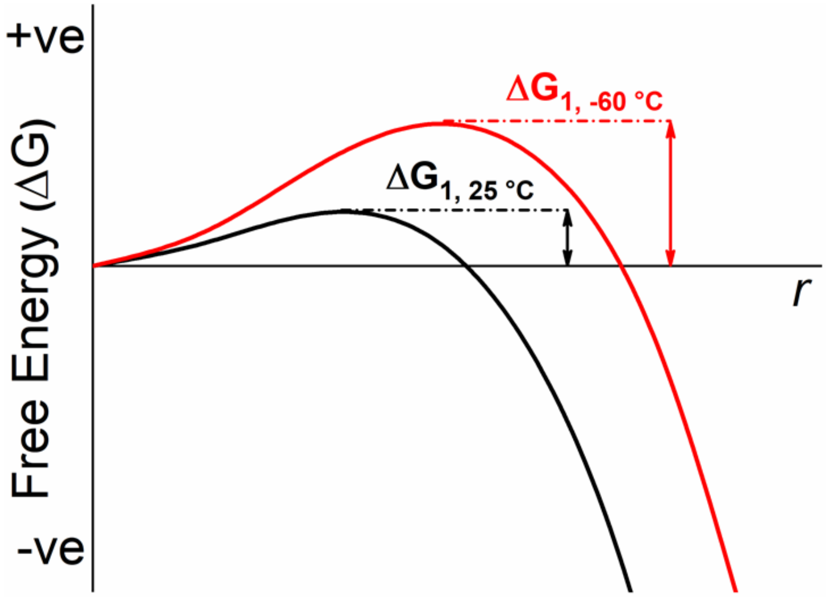

Supplementary Figure 1 Schematic for nucleation energy barriers vs. nucleation radius. The nucleation barrier height of reduced metal atoms can be significantly increased by lowering the temperature of solution reactions.

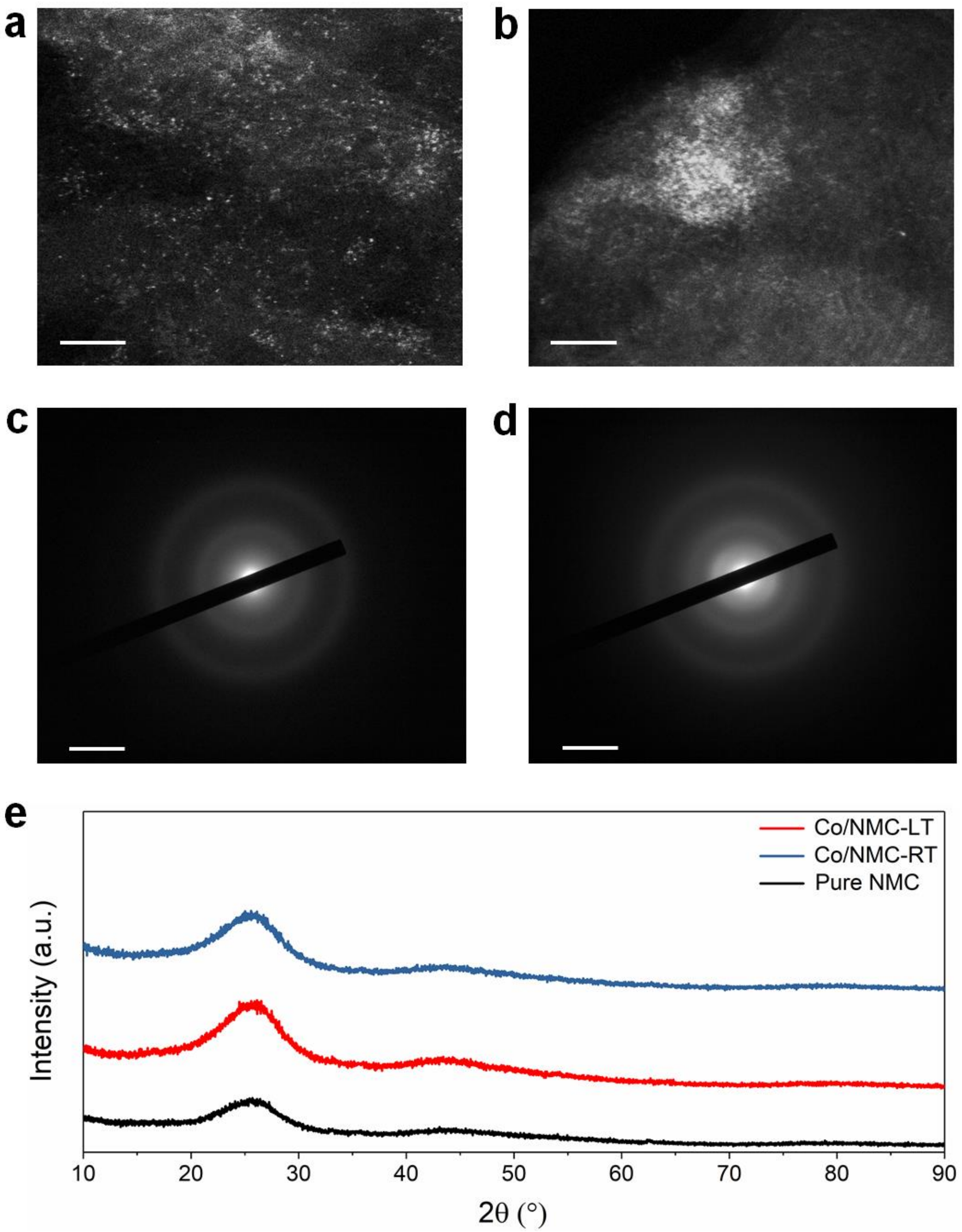

Supplementary Figure 2 Characterization and structures of relative samples. STEM images of (a) Co/NMC-LT and (b) Co/NMC-RT before annealing, scale bar: 2 nm. Electron diffraction images of (c) Co/NMC-LT and (d) Co/NMC-RT before annealing, scale bar: 5 1/nm. (e) XRD patterns of Co/NMC-LT, Co/NMC-RT, and pure NMC, no obvious Co or Co-based compounds peak can be identified.

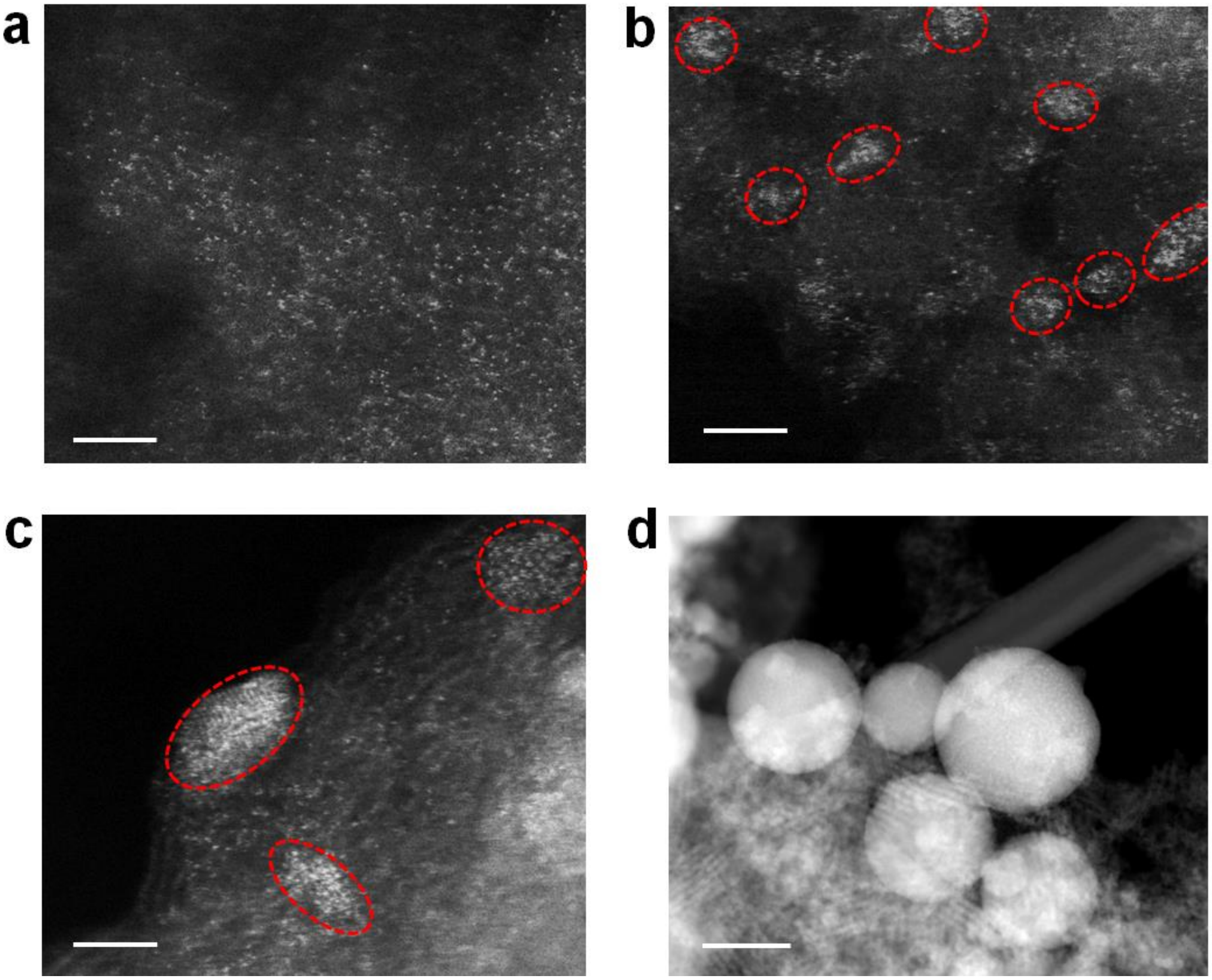

Supplementary Figure 3 STEM images of Co/NMC samples at different temperatures. (a) -60 ^{o}C (Co/NMC-LT), (b) -30 ^{o}C, (c) 25 ^{o}C (Co/NMC-RT) and (d) 60 ^{o}C, scale bar: 2 nm for (a-c), and 100 nm for (d).

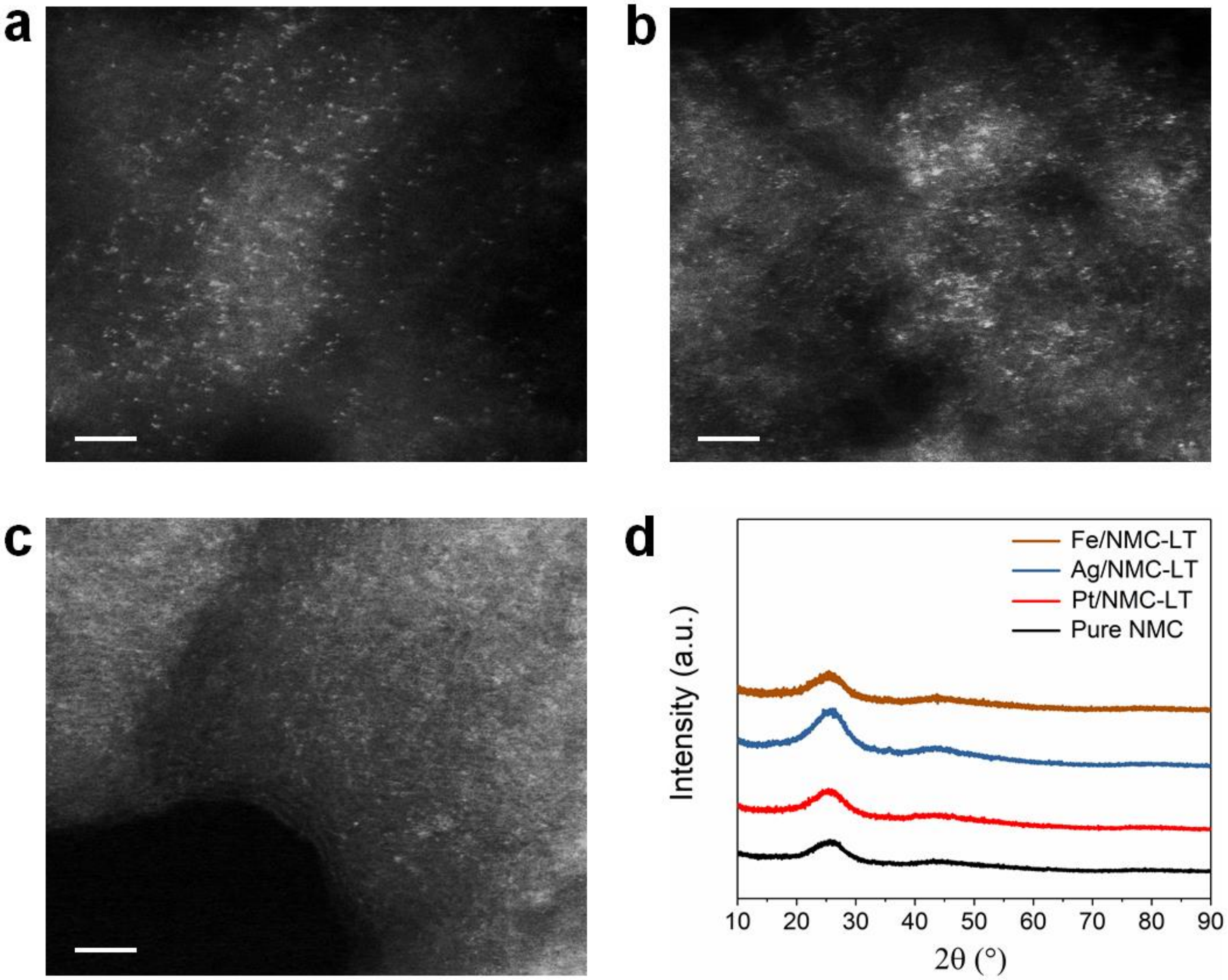

Supplementary Figure 4 Characterization and structures of different samples. STEM images of (a) Pt/NMC-LT, (b) Ag/NMC-LT, and (c) Fe/NMC-LT samples before annealing, scale bar: 2 nm for (a-c), and (d) corresponding XRD patterns, no obvious metal or metal-based compounds peak can be identified.

.

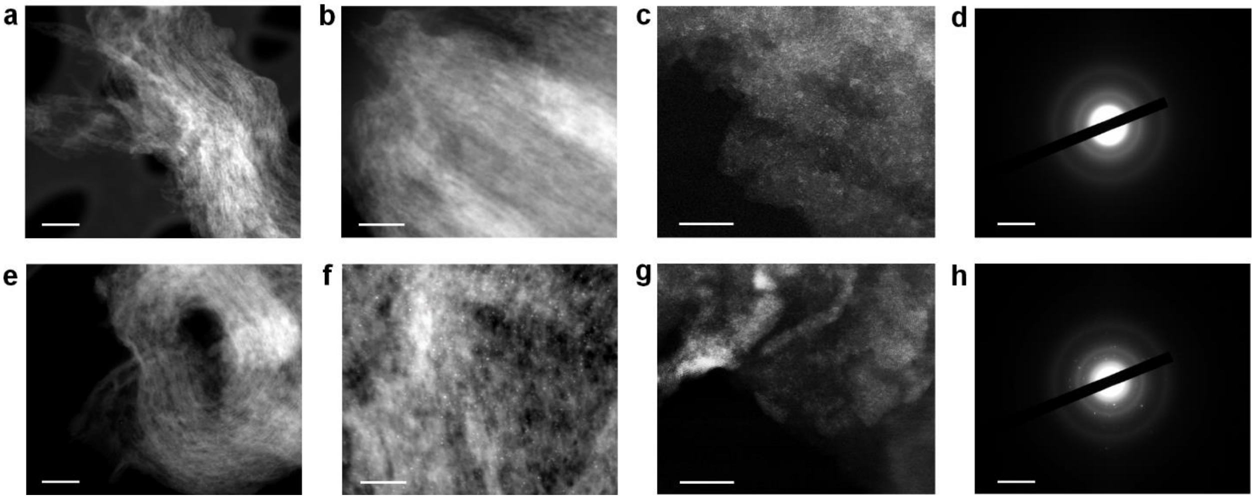

Supplementary Figure 5 STEM and electron diffraction images of different samples. (a-d) Co/NMC-LT900, and (e-f) Co/NMC-RT900 at lower magnifications, scale bar: 100 nm for (a, e), 50 nm for (b, f), 5 nm for (c, g) and 5 1/nm for (d, h). No obvious nanoparticles can be observed for Co/NMC-LT900.

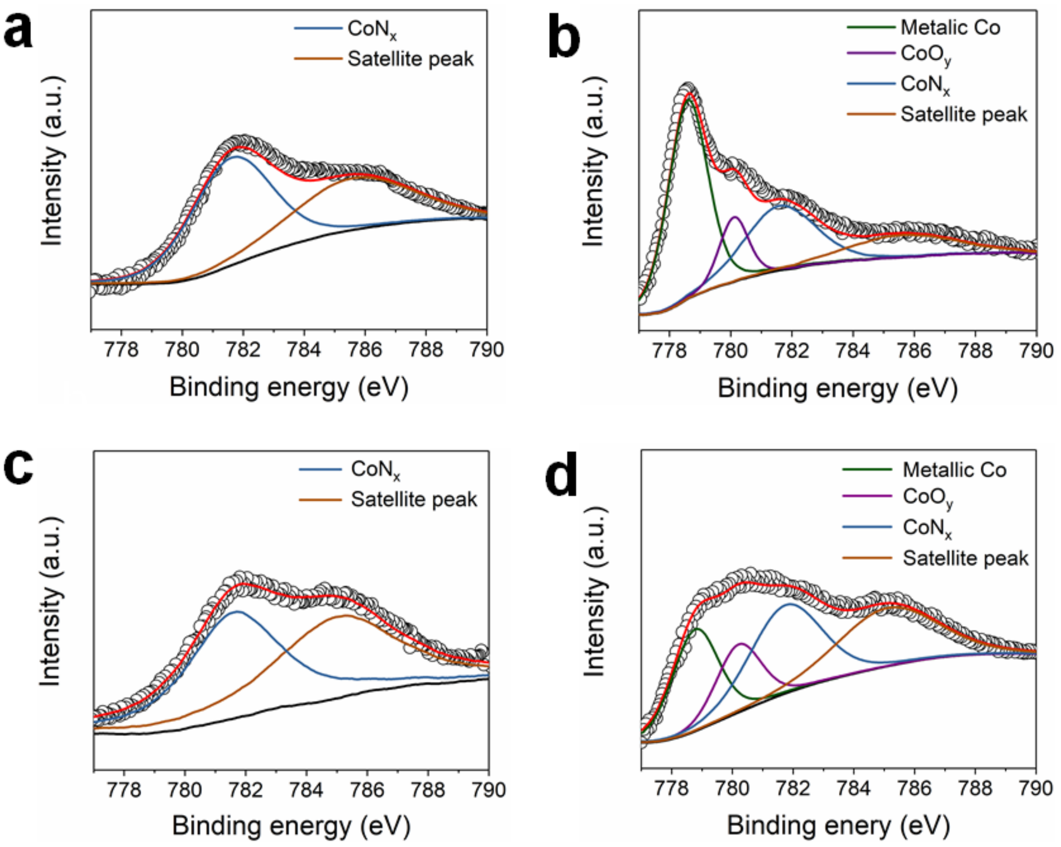

Supplementary Figure 6 Co 2p 3/2 high-resolution XPS spectra. (a) Co/NMC-LT900, (b) Co/NMC-RT900, (c) Co/NMC-LT and (d) Co/NMC-RT. Obvious metallic Co component for Co/NMC-RT900 can be identified after annealing at 900 ^{o}C, which indicates that some Co-N bonds may be broken and thus cause the formation of Co NPs.

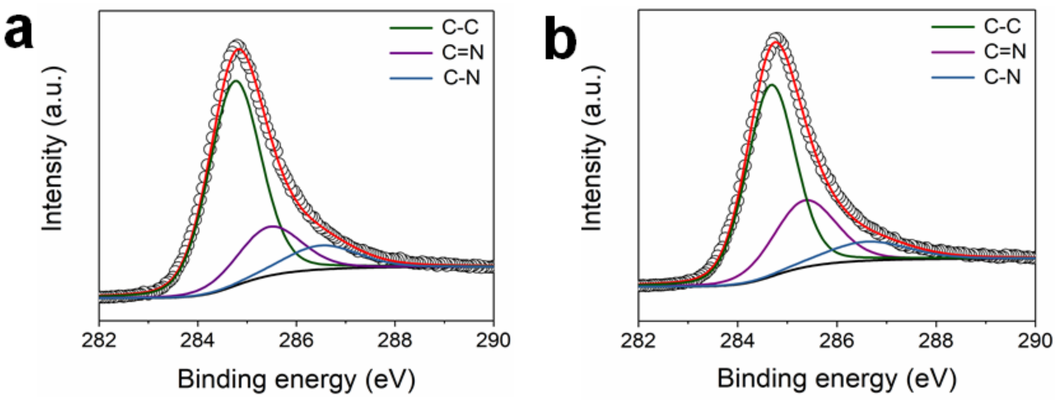

Supplementary Figure 7 C 1s high-resolution XPS spectra. (a) Co/NMC-LT900 and (b) Co/NMC-RT900.

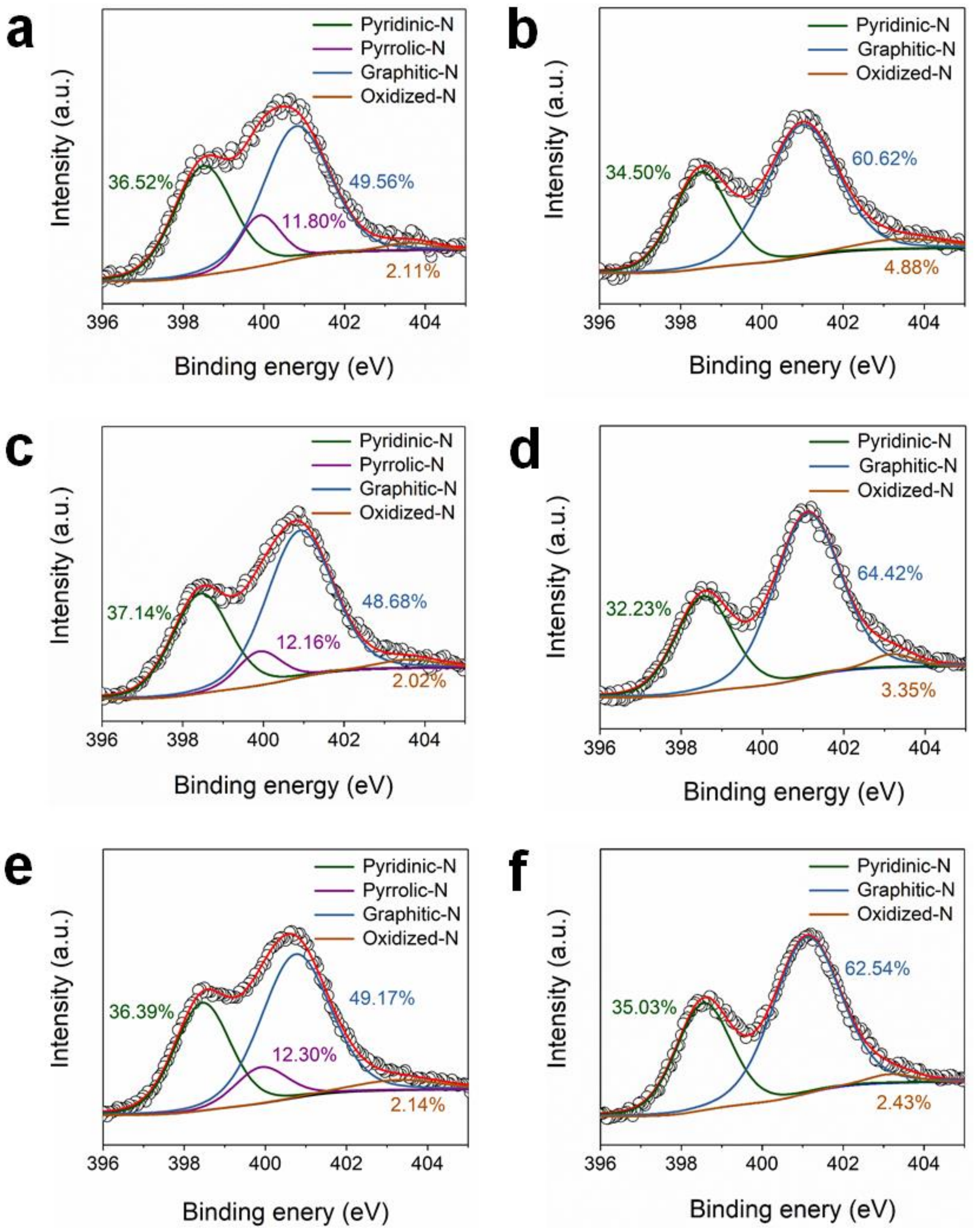

Supplementary Figure 8 N 1s high-resolution XPS spectra. (a) Co/NMC-LT, (b) Co/NMC-LT900, (c) Co/NMC-RT, (d) Co/NMC-RT900, (e) NMC, and (f) NMC-900. The decline in the total N content after annealing at 900oC can be attributed to the loss of five-sided pyrrolic N.

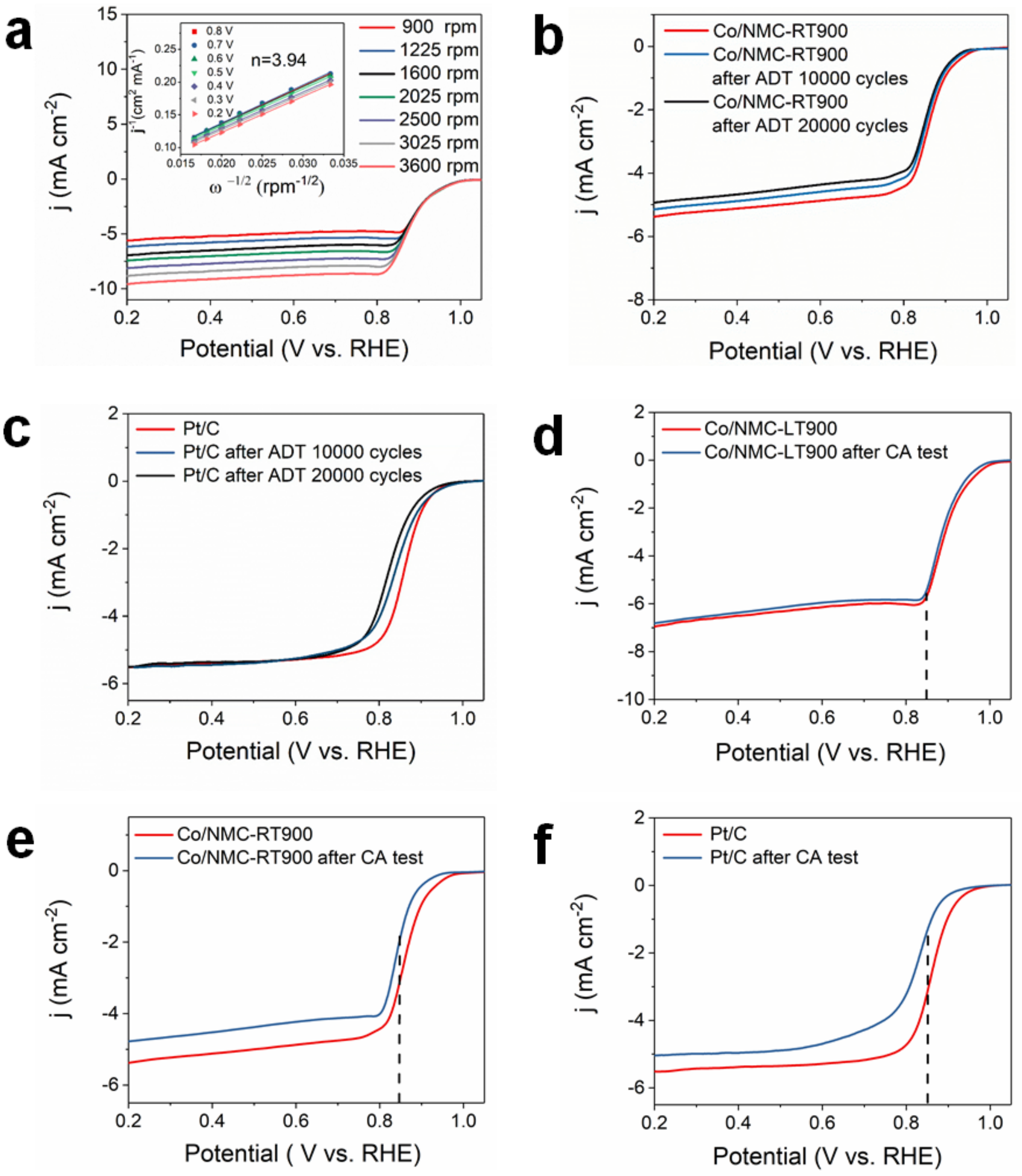

Supplementary Figure 9 ORR performance in O_2-saturated alkaline electrolyte. (a) RDE polarization curves of Co/NMC-LT900 at various rotation rates, inset: K-L plot of J^{-1} versus ω^{-1}. LSV curves of (b) Co/NMC-RT900 and (c) Pt/C before and after 10,000 and 20,000 potential cycles. LSV curves of (d) Co/NMC-LT900, (e) Co/NMC-LT900 and (f) Pt/C at 1600 rpm before and after a 27 h long-term stability test at the potential of 0.85 V vs. RHE in O_2-saturated 0.1 M KOH solution.

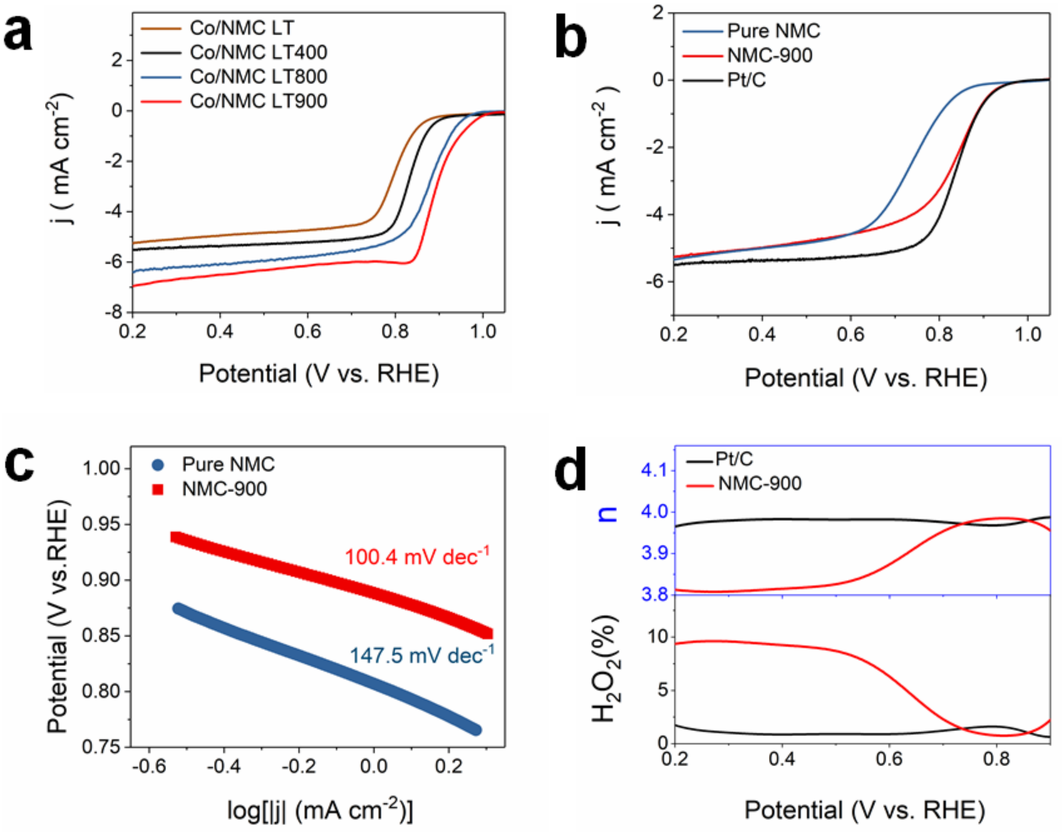

Supplementary Figure 10 ORR performance in O_2-saturated alkaline electrolyte. LSV curves of (a) Co/NMC-LT, Co/NMC-LT400, Co/NMC-LT800, and Co/NMC-LT900 (b) pure NMC, NMC-900, and Pt/C catalysts. (c) Tafel slope values of pure NMC and NMC-900 at low over-potential regions at a sweep rate of 10 mV s^{-1} and an electrode rotation speed of 1600 rpm. (d) electron transfer number n (top) and H_2O_2 yield (bottom) vs potential of Pt/C and NMC-900 in O_2-saturated 0.1 M KOH solution.

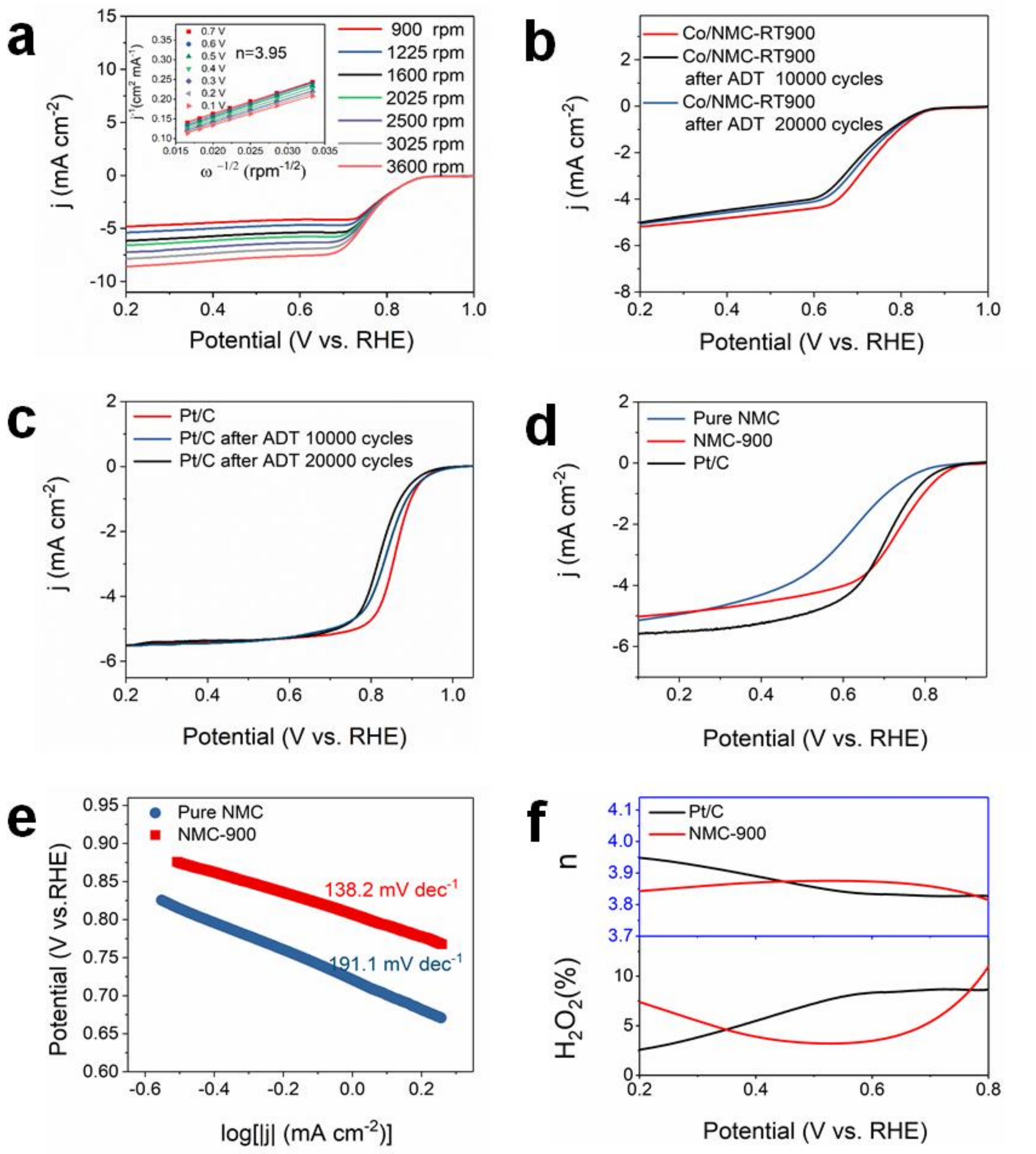

Supplementary Figure 11 Characterization and structures of different samples. (a) RDE polarization curves of Co/NMC-LT900 at various rotation rates, inset: K-L plot of J^{-1} versus ω^{-1}. LSV curves of (b) Co/NMC-RT900 and (c) Pt/C before and after 10,000 and 20,000 potential cycles. (d) LSV curves of pure NMC, NMC-900 and Pt/C catalysts. (e) Tafel slope values of pure NMC and NMC-900 at low over-potential regions at a sweep rate of 10 mV s^{-1} and an electrode rotation speed of 1600 rpm. (f) electron transfer number n (top) and H_2O_2 yield (bottom) vs potential of Pt/C and NMC-900 in O_2-saturated 0.05 M PBS solution.

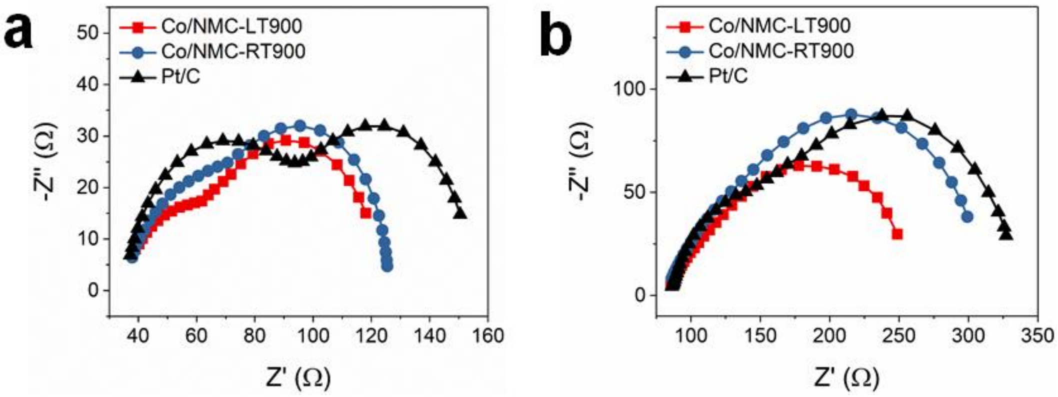

Supplementary Figure 12 Nyquist plots obtained from EIS measurements. (a) 0.85 V (vs. RHE) in O_2-saturated 0.1 M KOH solution and (b) 0.75 V (vs. RHE) in O_2-saturated 0.05 M PBS solution.

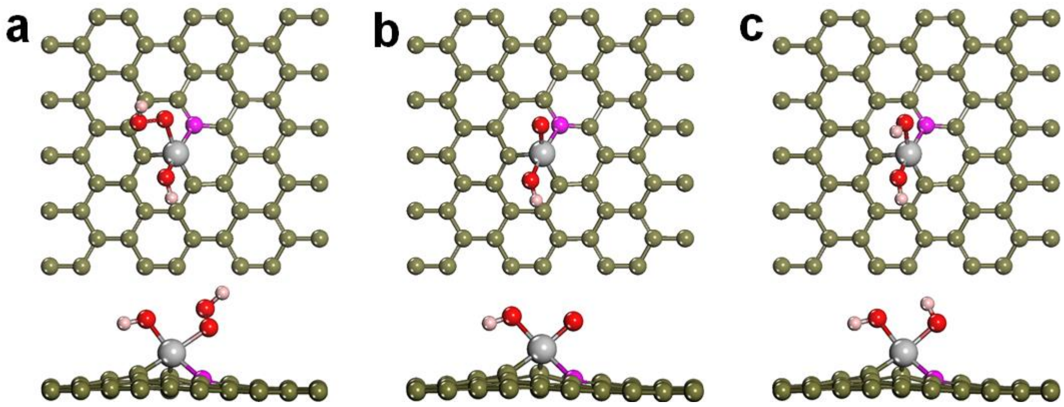

Supplementary Figure 13 Schematic model of ORR intermediates adsorption structures. (a) *OOH, (b) *O, and (c) *OH on the substrate of Co atoms adsorption on pyridinic-N, where the gray, olive, pink, red and light pink atoms represent Co, C, N, O and H atoms.

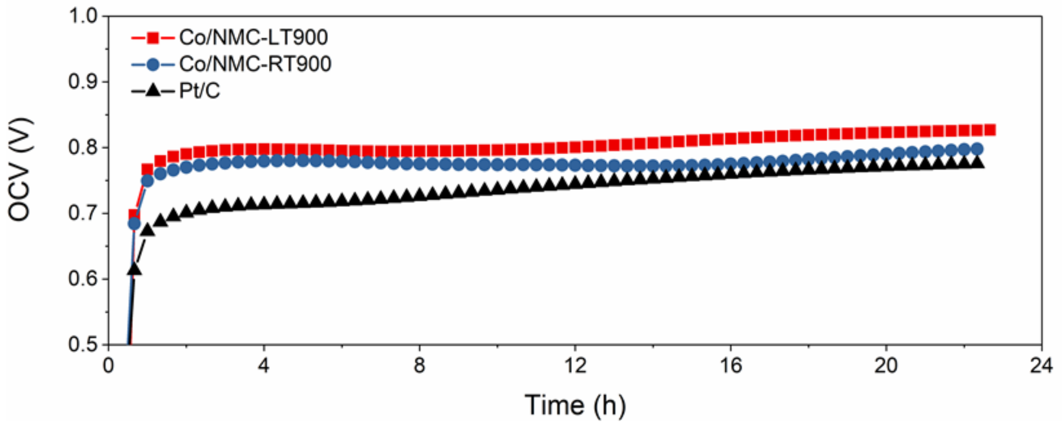

Supplementary Figure 14 Open circuit voltage (OCV) of MFCs. The steady OCV values were collected after running MFCs setups for 22 h without external resistance.

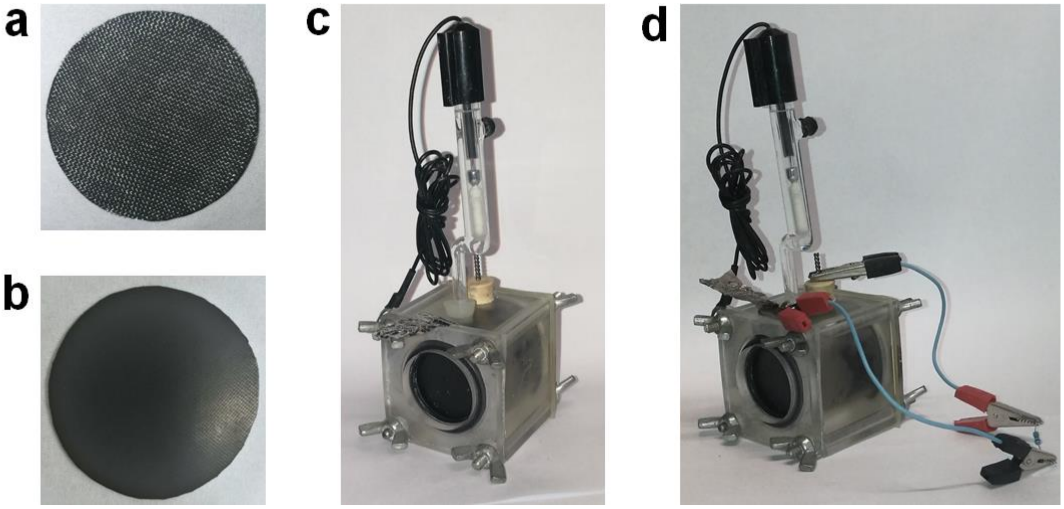

Supplementary Figure 15 Photographs of MFC setup. (a) the catalyst layer facing air and (b) the diffusion layer of cathode. (c, d) MFC setup without and with external resistance, respectively. All the photographs were took by the authors in lab and used for the first time.

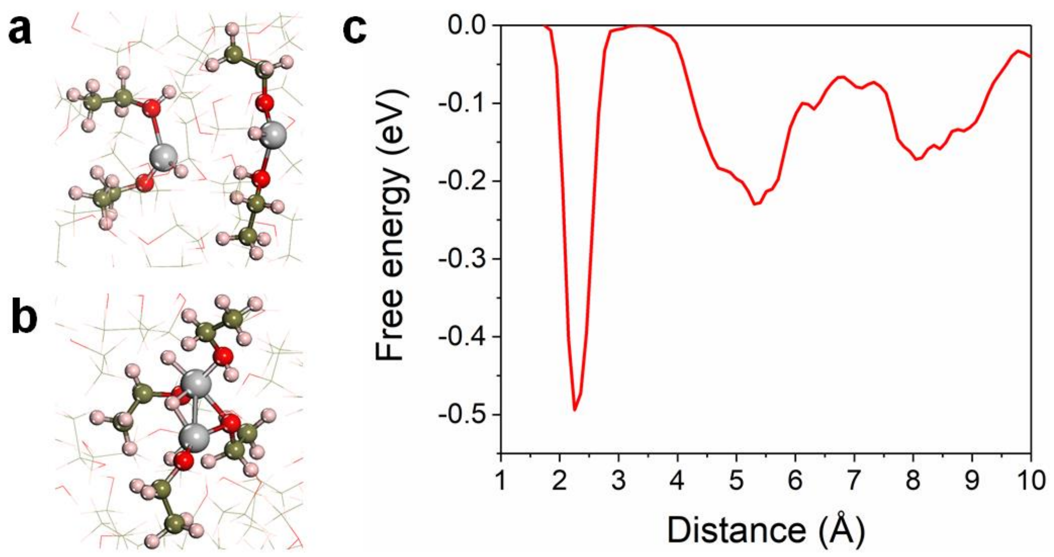

Supplementary Figure 16 The simulated atomic structures and free energy profile. For Co atoms in ethanol solvent by the first-principles molecular dynamics following by the metadynamics simulations. (a) Initial state with two single Co atoms and (b) final state with Co dimer. (c) Free energy profiles of dimer formation from two Co single atoms, where the initial distance between two single atoms are ~8.10 Å, and the Co single atoms first overcome a small energy barrier of 0.17 eV to an new intermediate state with a Co-Co distance of 5.40 Å. After this process, the Co single atoms further overcome an energy barrier of 0.24 eV to the final state of the Co dimer with a distance of 2.2 Å. It should be noted that the metadynamics simulation was terminated until the final state is more stable than the initial state, thus the free energy profile of final state was not fully constructed. Here, the ethanol molecules bind with Co atoms were highlighted by balls and bonds, and the other ethanol molecules were shown by bonds and sticks. The red, light pink, olive and gray atoms represent the O, H, C and the Co atoms, respectively.

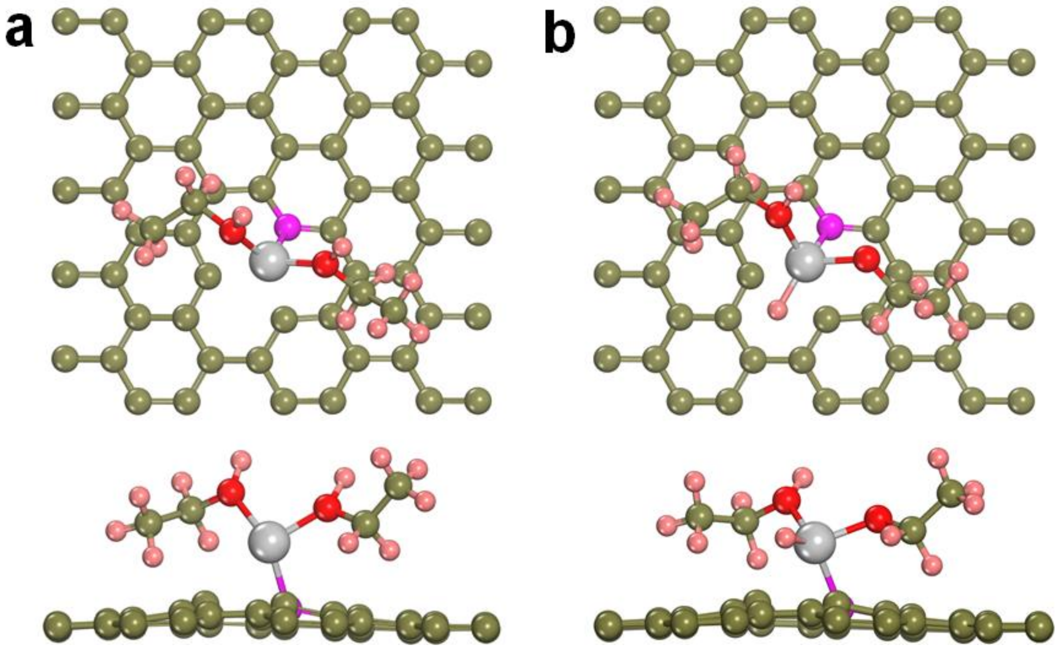

Supplementary Figure 17 Calculated top and side view of adsorption on Pyridinic-N. (a) molecular and (b) dissociated ethanol molecules. The gray, olive, pink, red and light pink atoms represent Co, C, N, O and H atoms.

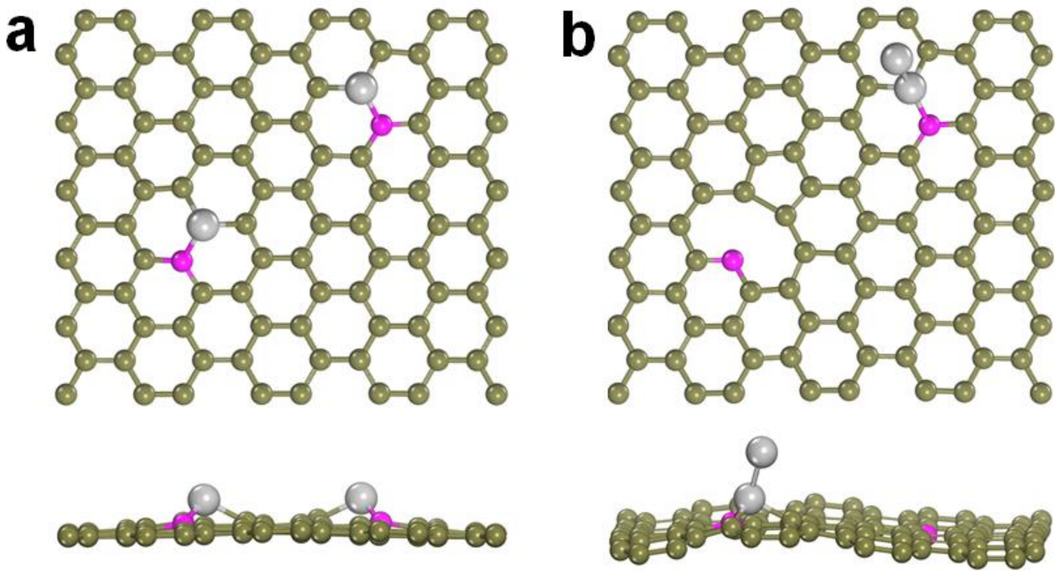

Supplementary Figure 18 Calculated top and side view of adsorption on Pyridinic-N. (a) two separated and (b) dimer Co atoms adsorption. The gray, olive and pink atoms represent Co, C and N atoms.

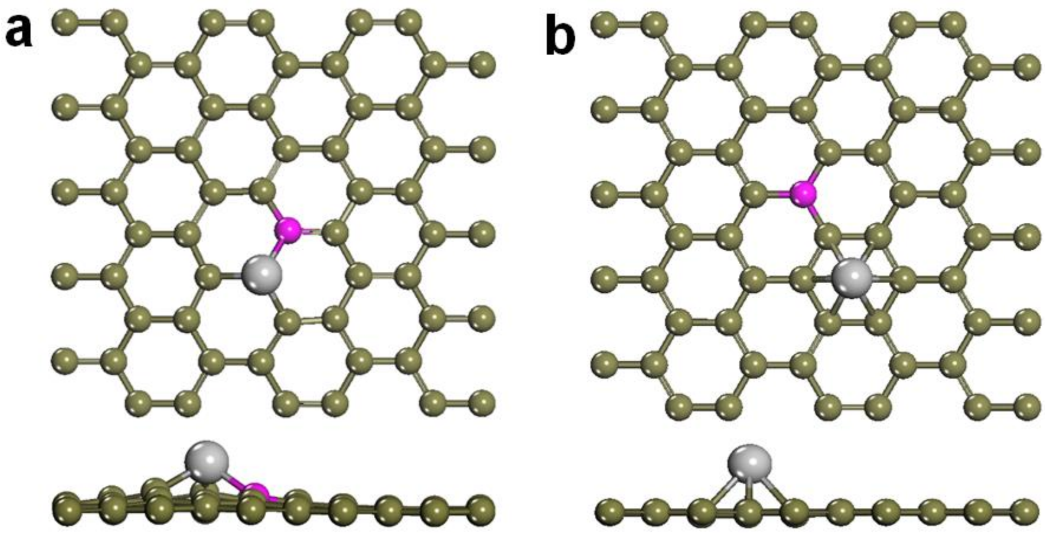

Supplementary Figure 19 Schematic model of Co atoms adsorption sites. (a) Isolated Co atom bound with pyridinic-N, and (b) Isolated Co atom bound with graphitic-N, where the gray, olive and pink atoms represent Co, C and N atoms.

Supplementary Table 1 Database of relative Enthalpies, Gibbs energies and entropies. Enthalpies and Gibbs energies of formation and entropies of the elements and inorganic compounds insolution synthesis of Co, Fe, Ag, and Pt [21]:

Substance	Physical state	$\Delta_f H^\circ$ kJ mol^{-1}	$\Delta_f G^\circ$ kJ mol^{-1}	S° J deg^{-1} mol^{-1}
$CoCl_2$	aq	-392.5	-316.7	0
N_2H_4 hydrazine	lq	50.6	149.3	121.2
KOH	aq	-482.37	-440.53	91.6
Co	c	0	0	30
N_2	g	0	0	191.609
KCl	aq	-419.53	-414.51	159.0
HCl	aq	-167.15	-131.25	56.5
H_2O	lq	-285.830	-237.14	69.95
$FeCl_2$	aq	-423.4	-341.3	-24.7
$AgNO_3$	aq	-101.80	-34.23	219.2
H_2PtCl_6	aq	-668.2	-482.8	220.1
NH_4NO_3	aq	-339.87	-190.71	259.8
Fe	c	0	0	27.32
Ag	c	0	0	42.55
Pt	c	0	41.63	25.87

Supplementary Table 2 XANES structural fitting parameters for samples. N, coordination number; R, distance between absorber and backscatter atoms; σ^2, the Debye-Waller factor value; The Fourier transformation of the k^3-weighted EXAFS oscillations, $k^3\ \chi(k)$, from k space to R space was performed over a range from 2.368 to 9.812 Å^{-1} (3.275-13.315 Å^{-1} for Co foil) to obtain a radial distribution function.

Sample	Path	N	R(Å)	σ^2 (10^{-3}Å^2)
Co/NMC-LT900	Co-N/C	4.1	1.98(6)	8.3
Co/NMC-RT900	Co-Co	9.7	2.49(7)	5.7
Co foil	Co-Co	12	2.49(3)	6.3

Supplementary Table 3 Comparison of the ORR activity. Between Co/NMC-LT900 electrocatalyst and recently reported transition metals-based nanocatalysts and atomically dispersed nonprecious metals catalysts in O_2-saturated 0.1 M KOH solution in literature.

Catalysts	Mass loading (mg cm^{-2})	E_{onset} (V vs. RHE)	$E_{1/2}$ (V vs. RHE)	J_k@0.85V J_k@0.80V (mA cm^{-2})	Reference
**Co/NMC-LT900**	**0.408**	**1.03**	**0.897**	**10.7** **46.8**	**This study**
Co_3O_4/N-rmGO	0.17	0.92	0.83	~2.14 @0.85 V	Ref. 22
Ag-Co	0.092	~0.91	~0.81	~0.36 @0.85 V ~1.43 @0.80 V	Ref. 23
NT-G (1.10 wt.% Fe)	0.485	~1.05	~0.88	--	Ref. 24
AuPdCo/C-intermetallic	0.08	~0.97	~0.86	--	Ref. 25
c-$CoMn_2$/C	0.18	0.95	0.83	3.58 @0.85 V	Ref. 26
SC CoO NRs	0.4	0.92	0.83	17.8 @0.6 V	Ref. 27
CPG-900	0.24	0.978	~0.87	--	Ref. 28
LT-$Li_{0.5}CoO_2$	~0.25	~0.9	~0.72	--	Ref. 29
Co@Co_3O_4@C-CM	0.10	0.93	0.81	--	Ref. 30
$Co_3(PO_4)_2$C-N/rGOA	0.25	0.962	0.837	38.46 @0.75V 45.41 @0.70V	Ref. 31
CuCo/NC	0.182	0.96	0.884	--	Ref. 32
Co SAS/N-C (900)	0.408	0.982	0.881	21.2 @0.80V	Ref. 33
Co-C_3N_4/CNT	0.408	~0.92	~0.85	--	Ref. 34
Co-N/CNFs	0.2	0.92	0.82	--	Ref. 35
Co-P,N-CNT	0.102	0.916	~0.80	--	Ref. 36

N/Co-doped PCP//NRGO	0.714	0.97	0.86	11.66 @0.7V	Ref. 37
Fe-N-SCCFs	0.24	1.03	0.883	--	Ref. 38
Fe-N-CNTAS-5-900	0.1	0.97	0.88	~40 @0.70V	Ref. 39
SA-Fe/NHPC	0.1	~0.98	0.87	4.1 @0.88V	Ref. 40
ZnN_x/BP	0.39	0 Vs. SCE	-0.175 Vs. SCE	--	Ref. 41

Supplementary Table 4 The fitted data of Nyquist plots. Corresponding equivalent circuits [$R_s(R_{ct1}Q_1)(R_2Q_2)$]. Rs is the solution resistance, the high-frequency (Rct_1, Q_1) element is associated with the charge-transfer process through electrode-electrolyte interface, and the low-frequency (R_2, Q_2) element can be attributed to the O_2 mass-transport.

Sample	0.1 M KOH solution			0.05 M PBS solution		
	$R_s(\Omega)$	$Rct_1(\Omega)$	$R_2(\Omega)$	$R_s(\Omega)$	$Rct_1(\Omega)$	$R_2(\Omega)$
Co/NMC-LT900	34.4	41.5	38.6	85.6	79.8	94.7
Co/NMC-RT900	34.6	52.9	45.2	86.2	105.8	109.1
Pt/C	34.2	62.5	56.4	86.4	92.4	126.2

Supplementary Table 5 Calculated ΔE_a of species on substrate, ZPE, and TS. The values for H_2O (l) and H_2 (g) are at T = 298 K.

Species	ΔE_a (eV)	ZPE (eV)	TS(eV)
*OOH	3.85	0.45	-
*O	2.96	0.10	-
*OH	0.66	0.35	-
H_2O(l)	-	0.58	0.67
H_2(g)	-	0.28	0.41

Supplementary Table 6 Calculated values of ΔG (*OOH), ΔG (*O), and ΔG (*OH). U = 0.00 and 0.90 V and the unit is given in eV.

U (V)	ΔG (*OOH)	ΔG (*O)	ΔG (*OH)
0.00	3.83	2.90	0.74
0.90	1.13	1.10	-0.16

Supplementary Table 7 Comparison of ORR catalysts for cathode of MFCs. The maximum power densities between Co/NMC-LT900 air-cathode and recently reported various catalysts in literature.

MPD: the maximum power density of MFCs; MCD: the maximum current density of MFCs; NaAc: sodium acetate.

Catalyst Category	Catalyst	MPD (mW m^{-2})	MPD of Pt/C (mW m^{-2})	MCD (A m^{-2})	MCD of Pt/C (A m^{-2})	Electrolyte	Anode Substrate	Cathode size (cm^2)	Catalyst loading (mg cm^{-2})	Reference
Non-noble metal-carbon and metal-nitrogen-carbon based materials	**Co/NMC-LT900**	**2550**	**1560**	**22.3**	**12.6**	**50 mM PBS**	**1 g L^{-1} NaAc**	**7.0**	**5.45**	**This study**
	CoNC-900	1660	1190	~8.8	~6.2	50 mM PBS	1 g L^{-1} Glucose	7.0	--	Ref. 42
	N-Fe/Fe_3C@C	1670	1550	~10.1	~8.0	100 mM PBS	1 g L^{-1} NaAc/Glucose	12.0	0.5	Ref. 43
	Fe-N-G	1149.8	561.1	~3.7	~3.9	50 mM PBS	0.5 g L^{-1} NaAc	19.6	0.5	Ref. 44
	Fe/Co/C/N	1200	500	~7.2	~1.75	200 mM PBS	Mixture of starch, peptone and fish extract	1.0	4.0	Ref. 45
	3D Fe-N-C	1218	788	~7.8	~5.7	10 mM PBS	Mixture of glucose and yeast extract	16.0	0.5	Ref. 46
	Fe-N-C	2437	--	~6.2	--	50 mM PBS	1 g L^{-1} NaAc	7.0	--	Ref. 47
	Fe/N-HCN	1300	1132	~6.4	~5.3	50 mM PBS	1 g L^{-1} NaAc	--	2.0	Ref. 48
	BNFe-C-G	1046.2	724.3	~4.4	~3.5	50 mM PBS	1g L^{-1} sucrose	7.0	--	Ref. 49
	Fe-BP(N)	2430	--	~16	--	100mM KPBS	50% activated sludge	2.85	2.0	Ref. 50
	Fe-N-C/AC	2600	--	11.8	--	50 mM	1 g L^{-1} NaAc	7.0	27.0	Ref. 51

	Co-N-C/AC	2000		~10.0		PBS				
	Na-N-C/AC	2100		~9.8						
	CoFe-PDAP	1100	500	~5.3	~1.8	PBS	Mixture of starch, peptone and fish extract	1.0	4.0	Ref. 52
	NiCo/NC	1693.6	984.7	~10.7	~8.2	10 mM PBS	Mixture of glucose and yeast extract	16.0	0.5	Ref. 53
	N-G @CoNi/BCNT	2000	2600	6.7	7.4	50 mM PBS	1 g L^{-1} NaAc	1.0	5.0	Ref. 54
	Fe-Ricobendazole	1950	~1600	~13	~11	100 mM K-PBS	2 g L^{-1} NaAc	2.8	2.0	Ref. 55
	Fe-Ricobendazole	2090	--	~13.0	--	100 mM K-PBS	3 g L^{-1} NaAc	--	2.0	Ref. 56
	FeEDTAAC_0.2	1580	1550	~7.9	~7.6	50 mM PBS	1 g L^{-1} NaAc	7.0	5.0	Ref. 57
	Fe-AAPyr	1670	1130	10.0	~9.2	50 mM PBS	1 g L^{-1} NaAc	2.9	2.1	Ref. 58
	Fe-AAPyr-5	2130	--	14.0	--	100 mM KPBS	50% activated sludge	2.8	5.0	Ref. 59
	$Cu_3(BTC)_2$	1772	--	~4.7	--	50 mM PBS	2 g L^{-1} NaAc	7.0	--	Ref. 60
Non-noble metal oxides based materials	C-CoO_x-FePc	654	~830	~4.2	~3.3	50 mM PBS	1 g L^{-1} NaAc	7.0	4.0	Ref. 61
	CoO@N-AC	1650.1	1201.4	5.5	4.5	50 mM PBS	2 g L^{-1} acetate	7.0	--	Ref. 62
	CoO_x/CoP	1914.4	--	~7.3	--	100 mM PBS	--	--	--	Ref. 63
	MnO_2 AC	1554	--	~4.75	--	50 mM PBS	1 g L^{-1} NaAc	7.0	--	Ref.64
	N-type Cu_2O doped AC	1390	--	~5.3	--	50 mM PBS	1 g L^{-1} NaAc	7.0	--	Ref. 65
	Fe_3O_4/PGC-CS	1443	1192	~5.5	~4.9	50 mM PBS	1 g L^{-1} NaAc/Glucose	7.0	5.0	Ref. 66
	$CoFe_2O_4$@N-	1770.8	1201	~7.0	~4.5	50 mM	2 g L^{-1} acetate	7.0	--	Ref. 67

	AC					PBS				
	$NiCo_2O_4$-CFC	645	571	~4.0	~4.0	100 mM PBS	1g L^{-1} Sucrose	7.0	--	Ref. 68
	NiO/CNT	670	--	2.7	--	50 mM PBS	1 g L^{-1} Glucose	3.14	0.5	Ref. 69
Carbon based materials	CNF_{1000}	1747	--	~5.4	--	M9 buffer	Mixture of peptone and yeast extract	--	--	Ref. 70
	NDC	2300	1330	~11.5	8.8	50 mM PBS	1 g L^{-1} NaAc	7.0	9.0	Ref. 71
	N-CNT	1600	1393	~6.1	~6.0	50 mM PBS	1 g L^{-1} NaAc	--	0.5	Ref. 72
	N-graphene	1350	1420	~5.8	~6.0	50 mM PBS	1 g L^{-1} NaAc	--	0.5	Ref. 73
	N-graphene	776	750	~2.55	~2.55	100 mM PBS	1 g L^{-1} NaAc	9.0	2.0	Ref. 74
	P-carbon	1312	1220	~4.7	~5.3	50 mM PBS	1 g L^{-1} NaAc	7.0	0.5	Ref. 75
	N,P-carbon	2293	1680	~9.7	~8.1	50 mM PBS	1 g L^{-1} NaAc	7.0	20.0	Ref. 76
	BC-HT-NP-HT	1719	1039	11.5	~8.0	50 mM PBS	2.04 g L^{-1} NaAc	7.0	1.4	Ref. 77
	AC+CB	1560	570	~10.0	~5.0	50 mM PBS	1 g L^{-1} NaAc	7.0	42.9	Ref. 78
Noble metal based materials	Pt	1000-1500	--	8-12	--	50 mM PBS	1 g L^{-1} NaAc	--	--	In most cases as listed references
	CNT-texitile-Pt	837	391	5.2	4.0	100 mM PBS	1 g L^{-1} Glucose	2.0	--	Ref. 79
	Co-N-C/Pt	1008	779	~4.7	~3.2	50 mM PBS	1 g L^{-1} Sucrose	7.0	4.0	Ref. 80
	Ag/FeS/PGC	1361	483	4.4	2.6	50 mM PBS	1 g L^{-1} Glucose	7.0	--	Ref. 81
	Ag-50 AC	1080	--	~4.5	--	50 mM PBS	1 g L^{-1} NaAc	7.0	--	Ref. 82

Supplementary References

1. Yang, T.-H., Gilroy, K. D., & Xia, Y. N., Reduction rate as a quantitative knob for achieving deterministic synthesis of colloidal metal nanocrystals. *Chem. Sci.* **8**, 6730-6749 (2017).
2. Hsu, S. C. *et al.* Turning the halide switch in the synthesis of Au-Pd alloy and core-shell nanoicosahedra with terraced shells: Performance in electrochemical and plasmon-enhanced catalysis. *Nano Lett.* **16**, 5514-5520 (2016).
3. Zhou, M. *et al.* Quantitative Analysis of the Reduction Kinetics Responsible for the One-Pot Synthesis of Pd-Pt Bimetallic Nanocrystals with Different Structures. *J. Am. Chem. Soc.* **138**, 12263-12270 (2016).
4. Liu, Q., et al. Quantifying the Nucleation and Growth Kinetics of Microwave Nanochemistry Enabled by in Situ High-Energy X-Ray Scattering. *Nano Lett.* **16**, 715-720 (2015).
5. Pacławski, K., & Sak, T. Kinetics and mechanism of the reaction of gold (III) chloride complexes with formic acid. *J. Min. Metall. B* **51**, 133-142 (2015).
6. Kwon, S. G. & Hyeon, T., Formation mechanisms of uniform nanocrystals via hot-injection and heat-up methods. *Small* **7**, 2685-2702 (2011).
7. Yu, H. et al. The XAFS beamline of SSRF. *Nucl. Sci. Tech.* **26**, 050102 (2015).
8. Newville, M. IFEFFIT: interactive XAFS analysis and FEFF fitting. *J. Synchrotron Rad.* **8**, 322-324 (2001).
9. Dong, H. *et al.* Hydrogen Peroxide Generation in Microbial Fuel Cells Using graphene-based air-cathodes. *Bioresource Technol.* **247**, 684-689 (2017).
10. Lovley, D. R., Phillips, E. J. P. General Microbial Ecology Novel Mode of Microbial Energy Metabolism: Organic Carbon Oxidation Coupled to Dissimilatory Reduction of Iron or Manganese. *Appl. Environ. Microbiol.* **54**, 1472-1480 (1988).
11. Logan, B. E. *et al.* Microbial fuel cells: methodology and technology. *Environ. Sci. Technol.* **40**, 5181-5192 (2006).
12. Kresse, G. & Hafner, J. Ab initiomolecular dynamics for liquid metals. *Phys. Rev. B* **47**, 558-561, (1993).
13. Kresse, G. & Furthmüller, J. Efficient iterative schemes forab initiototal-energy calculations using a plane-wave basis set. *Phys. Rev. B* **54**, 11169-11186 (1996).
14. Blöchl, P. E. Projector augmented-wave method. *Phys. Rev. B* **50**, 17953-17979, (1994).
15. Kresse, G. & Joubert, D., From ultrasoft pseudopotentials to the projector augmented-wave method. *Phys. Rev. B* **59**, 1758-1775 (1999).
16. Monkhorst, H. J. & Pack, J. D. Special points for Brillouin-zone integrations. *Phys. Rev. B* **13**, 5188-5192, (1976).
17. VandeVondele, J. et al. Quickstep: Fast and accurate density functional calculations using a mixed Gaussian and plane waves approach. *Comput. Phys. Commun.* **167**, 103-128, (2005).
18. Vandevondele, J. & Hutter, J. Gaussian basis sets for accurate calculations on molecular systems in gas and condensed phases. *J. Chem. Phys.* **127**, 114105 (2007).
19. Goedecker, S., Teter, M. & Hutter, J. Separable dual-space Gaussian pseudopotentials. P*hys. Rev. B* **54**, 1703-1710 (1996).
20. Kolafa J. Time-reversible always stable predictor-corrector method for molecular dynamics of polarizable molecules. *J. Comput. Chem.* **25**, 335-342 (2004).
21. Speight, J. G., Lange's handbook of chemistry. Vol. *1*. New York: McGraw-Hill, (2005).
22. Liang, Y. et al. Co_3O_4 nanocrystals on graphene as a synergistic catalyst for oxygen reduction reaction. *Nat. Mater.* **10**, 780 (2011).

23. Holewinski, A., Idrobo, J. C., & Linic, S. High-performance Ag-Co alloy catalysts for electrochemical oxygen reduction. *Nat. Chem.* **6**, 828 (2014).
24. Li, Y. et al. An oxygen reduction electrocatalyst based on carbon nanotube-graphene complexes. *Nat. Nanotech.* **7**, 394 (2012).
25. Kuttiyiel, K. A. et al. Gold-promoted structurally ordered intermetallic palladium cobalt nanoparticles for the oxygen reduction reaction. *Nat. Commun.* **5**, 5185 (2014).
26. Li, C. et al. Phase and composition controllable synthesis of cobalt manganese spinel nanoparticles towards efficient oxygen electrocatalysis. *Nat. Commun.* **6**, 7345 (2015).
27. Ling, T. et al. Engineering surface atomic structure of single-crystal cobalt (II) oxide nanorods for superior electrocatalysis. *Nat. Commun.* **7**, 12876 (2016).
28. Wang, J., Wang, K., Wang, F. B., & Xia, X. H. Bioinspired copper catalyst effective for both reduction and evolution of oxygen. *Nat. Commun.* **5**, 5285 (2014).
29. Maiyalagan, T., Jarvis, K. A., Therese, S., Ferreira, P. J., & Manthiram, A. Spinel-type lithium cobalt oxide as a bifunctional electrocatalyst for the oxygen evolution and oxygen reduction reactions. *Nat. Commun.* **5**, 3949 (2014).
30. Xia, W., Zou, R., An, L., Xia, D., & Guo, S. A metal-organic framework route to in situ encapsulation of Co@Co_3O_4@C core@ bishell nanoparticles into a highly ordered porous carbon matrix for oxygen reduction. *Energy Environ. Sci.* **8**, 568-576 (2015).
31. Zhou, T. et al. Nitrogen-doped cobalt phosphate@nanocarbon hybrids for efficient electrocatalytic oxygen reduction. *Energy Environ. Sci.* **9**, 2563-2570 (2016).
32. Kuang, M., Wang, Q., Han, P., & Zheng, G. Cu, Co-embedded N-enriched mesoporous carbon for efficient oxygen reduction and hydrogen evolution reactions. *Adv. Energy Mater.* **7**, 1700193 (2017).
33. Yin, P. et al. Single cobalt atoms with precise N-coordination as superior oxygen reduction reaction catalysts. *Angew. Che. Int. Ed.* **55**, 10800-10805 (2016).
34. Zheng, Y. Molecule-level g-C_3N_4 coordinated transition metals as a new class of electrocatalysts for oxygen electrode reactions. *J. Am. Chem. Soc.* **139**, 3336-3339 (2017).
35. Cheng, Q. et al. Single cobalt atom and N co-doped carbon nanofibers as highly durable electrocatalyst for oxygen reduction reaction. *ACS Cata.* **7**, 6864-6871 (2017).
36. Guo, S. et al. Atomic-scaled cobalt encapsulated in P, N-doped carbon sheaths over carbon nanotubes for enhanced oxygen reduction electrocatalysis under acidic and alkaline media. *Chem. Commun.* **53**, 9862-9865 (2017).
37. Hou, Y. et al. An advanced nitrogen-doped graphene/cobalt-embedded porous carbon polyhedron hybrid for efficient catalysis of oxygen reduction and water splitting. *Adv. Funct. Mater.* **25**, 872-882 (2015).
38. Wang, B. et al. Simple-cubic carbon frameworks with atomically dispersed iron dopants toward high-efficiency oxygen reduction. *Nano Lett.* **17**, 2003-2009 (2017).
39. Zhu, C. et al. Self-assembled Fe-N-doped carbon nanotube aerogels with single-atom catalyst feature as high-efficiency oxygen reduction electrocatalysts. *Small* **13**, 1603407 (2017).
40. Zhang, Z., Gao, X., Dou, M., Ji, J., & Wang, F. Biomass derived N-doped porous carbon supported single Fe atoms as superior electrocatalysts for oxygen reduction. *Small* **13**, 1604290 (2017).
41. Song, P. et al. Zn single atom catalyst for highly efficient oxygen reduction reaction. *Adv. Funct. Mater.* **27**, 1700802 (2017).
42. You, S. et al. Enhanced cathodic oxygen reduction and power production of microbial fuel cell based on noble-metal-free electrocatalyst derived from metal-organic frameworks. *Adv.*

Energy Mate. **6**, 1501497 (2016).
43. Wen, Z. et al. Nitrogen-enriched core-shell structured Fe/Fe_3C-C nanorods as advanced electrocatalysts for oxygen reduction reaction. *Adv. Mater.* **24**, 1399-1404 (2012).
44. Li, S. et al. Iron-and nitrogen-functionalized graphene as a non-precious metal catalyst for enhanced oxygen reduction in an air-cathode microbial fuel cell. *J. Power Sources* **213**, 265-269 (2012).
45. Zhao, Y., Watanabe, K., & Hashimoto, K. Efficient oxygen reduction by a Fe/Co/C/N nano-porous catalyst in neutral media. *J. Mater. Chem. A* **1**, 1450-1456 (2013).
46. Tang, H. et al. Iron-embedded nitrogen doped carbon frameworks as robust catalyst for oxygen reduction reaction in microbial fuel cells. *Appl. Catal. B-Environ.* **202**, 550-556 (2017).
47. Pan, Y. et al. Iron-nitrogen-activated carbon as cathode catalyst to improve the power generation of single-chamber air-cathode microbial fuel cells. *Bioresource Technol.* **206**, 285-289 (2016).
48. Zhou, L. et al. Soft-template assisted synthesis of Fe/N-doped hollow carbon nanospheres as advanced electrocatalysts for the oxygen reduction reaction in microbial fuel cells. *J Mater. Chem. A* **5**, 19343-19350 (2017).
49. Cao, C., Wei, L., Wang, G., & Shen, J. Superiority of boron, nitrogen and iron ternary doped carbonized graphene oxide-based catalysts for oxygen reduction in microbial fuel cells. *Nanoscale* **9**, 3537-3546 (2017).
50. Santoro, C. et al. Design of iron (II) phthalocyanine-derived oxygen reduction electrocatalysts for high-power-density microbial fuel cells. *ChemSusChem* **10**, 3243-3251 (2017).
51. Yang, W., & Logan, B. E. Immobilization of a metal-nitrogen-arbon catalyst on activated carbon with enhanced cathode performance in microbial fuel cells. *ChemSusChem* **9**, 2226-2232 (2016).
52. Zhao, Y., Watanabe, K., & Hashimoto, K. Self-supporting oxygen reduction electrocatalysts made from a nitrogen-rich network polymer. *J. Am. Chem. Soc.* **134**, 19528-19531 (2012).
53. Tang, H. et al. Metal-organic-framework-derived dual metal-and nitrogen-doped carbon as efficient and robust oxygen reduction reaction catalysts for microbial fuel cells. *Adv. Sci.* **3**, 1500265 (2016).
54. Hou, Y. et al. Nitrogen-doped graphene/CoNi alloy encased within bamboo-like carbon nanotube hybrids as cathode catalysts in microbial fuel cells. *J. Power Sources* **307**, 561-568 (2016).
55. Santoro, C. et al. Iron based catalysts from novel low-cost organic precursors for enhanced oxygen reduction reaction in neutral media microbial fuel cells. *Energy Environ. Sci.* **9**, 2346-2353 (2016).
56. Santoro, C. et al. A family of Fe-NC oxygen reduction electrocatalysts for microbial fuel cell (MFC) application: relationships between surface chemistry and performances. *Appl. Cata. B-Environ.* **205**, 24-33 (2017).
57. Xia, X. et al. Use of pyrolyzed iron ethylenediaminetetraacetic acid modified activated carbon as air-cathode catalyst in microbial fuel cells. *ACS Appl. Mater. Interfaces* **5**, 7862-7866 (2013).
58. Santoro, C. et al. High catalytic activity and pollutants resistivity using Fe-AAPyr cathode catalyst for microbial fuel cell application. *Sci. Rep.* **5**, 16596 (2015).
59. Santoro, C. et al. Power generation in microbial fuel cells using platinum group metal-free cathode catalyst: effect of the catalyst loading on performance and costs. *J. power sources*

378, 169-175 (2018).
60. Tian, P. et al. Porous metal-organic framework $Cu_3(BTC)_2$ as catalyst used in air-cathode for high performance of microbial fuel cell. *Bioresource Technol.* **244**, 206-212 (2017).
61. Ahmed, J., Yuan, Y., Zhou, L., & Kim, S. Carbon supported cobalt oxide nanoparticles-iron phthalocyanine as alternative cathode catalyst for oxygen reduction in microbial fuel cells. *J. Power Sources* **208**, 170-175 (2012).
62. Huang, Q., Zhou, P., Yang, H., Zhu, L., & Wu, H. CoO nanosheets in situ grown on nitrogen-doped activated carbon as an effective cathodic electrocatalyst for oxygen reduction reaction in microbial fuel cells. *Electrochim. Acta* **232**, 339-347 (2017).
63. Yang, T. et al. Surface-oxidized cobalt phosphide used as high efficient electrocatalyst in activated carbon air-cathode microbial fuel cell. *J. Power Sources* **363**, 87-94 (2017).
64. Zhang, P., Li, K., & Liu, X. Carnation-like MnO_2 modified activated carbon air cathode improve power generation in microbial fuel cells. *J. power sources* **264**, 248-253 (2014).
65. Zhang, X., Li, K., Yan, P., Liu, Z., & Pu, L. N-type Cu_2O doped activated carbon as catalyst for improving power generation of air cathode microbial fuel cells. *Bioresource Technol.* **187**, 299-304 (2015).
66. Ma, M. et al. Synthesis of iron oxide/partly graphitized carbon composites as a high-efficiency and low-cost cathode catalyst for microbial fuel cells. *ACS Appl. Mater. Interfaces* **6**, 13438-13447 (2014).
67. Huang, Q., Zhou, P., Yang, H., Zhu, L., & Wu, H. In situ generation of inverse spinel $CoFe_2O_4$ nanoparticles onto nitrogen-doped activated carbon for an effective cathode electrocatalyst of microbial fuel cells. *Chem. Eng. J.* **325**, 466-473 (2017).
68. Cao, C., Wei, L., Wang, G., & Shen, J. In-situ growing $NiCo_2O_4$ nanoplatelets on carbon cloth as binder-free catalyst air-cathode for high-performance microbial fuel cells. *Electrochim. Acta* **231**, 609-616 (2017).
69. Huang, J. et al. Nickel oxide and carbon nanotube composite (NiO/CNT) as a novel cathode non-precious metal catalyst in microbial fuel cells. *Biosens. Bioelectron.* **72**, 332-339 (2015).
70. Zou, L. et al. Tailoring unique mesopores of hierarchically porous structures for fast direct electrochemistry in microbial fuel cells. *Adv. Energy Mater.* **6**, 1501535 (2016).
71. Zhang, X. et al. High-performance carbon aerogel air cathodes for microbial fuel cells. *ChemSusChem* **9**, 2788-2795 (2016).
72. Feng, L., Yan, Y., Chen, Y., & Wang, L. Nitrogen-doped carbon nanotubes as efficient and durable metal-free cathodic catalysts for oxygen reduction in microbial fuel cells. *Energy Environ. Sci.* **4**, 1892-1899 (2011).
73. Feng, L., Chen, Y., & Chen, L. Easy-to-operate and low-temperature synthesis of gram-scale nitrogen-doped graphene and its application as cathode catalyst in microbial fuel cells. *ACS Nano* **5**, 9611-9618 (2011).
74. Liu, Y., Liu, H., Wang, C., Hou, S. X., & Yang, N. Sustainable energy recovery in wastewater treatment by microbial fuel cells: stable power generation with nitrogen-doped graphene cathode. *Environ. Sci. Technol.* **47**, 13889-13895 (2013).
75. Liu, Q. et al. Phosphorus-doped carbon derived from cellulose phosphate as efficient catalyst for air-cathode in microbial fuel cells. *J. Power Sources* **261**, 245-248 (2014).
76. Liu, Q. et al. Cellulose-derived nitrogen and phosphorus dual-doped carbon as high performance oxygen reduction catalyst in microbial fuel cell. *J. Power Sources* **273**, 1189-1193 (2015).
77. Yang, W., Li, J., Ye, D., Zhu, X., & Liao, Q. Bamboo charcoal as a cost-effective catalyst for

an air-cathode of microbial fuel cells. *Electrochim. Acta* **224**, 585-592 (2017).

78. Zhang, X., Xia, X., Ivanov, I., Huang, X., & Logan, B. E. Enhanced activated carbon cathode performance for microbial fuel cell by blending carbon black. *Environ. Sci. Technol.* **48**, 2075-2081 (2014).

79. Xie, X. et al. Nano-structured textiles as high-performance aqueous cathodes for microbial fuel cells. *Energy Environ. Sci.* **4**, 1293-1297 (2011).

80. Cao, C. et al. Gas-flow tailoring fabrication of graphene-like Co-N_x-C nanosheet supported sub-10 nm PtCo nanoalloys as synergistic catalyst for air-cathode microbial fuel cells. *ACS Appl. Mater. Interfaces* **9**, 22465-22475 (2017).

81. Sun, Y. et al. Biofouling inhibition on nano-silver/ferrous sulfide/partly-graphitized carbon cathode with enhanced catalytic activity and durability for microbial fuel cells. *Carbon* **119**, 394-402 (2017).

82. Pu, L. et al. Silver electrodeposition on the activated carbon air cathode for performance improvement in microbial fuel cells. *J Power Sources* **268**, 476-481 (2014).
